# Phylogenetic mapping of scale nanostructure diversity in snakes

**DOI:** 10.1186/s12862-019-1411-6

**Published:** 2019-04-16

**Authors:** Marcelle I. Arrigo, Luis M. De Oliveira Vilaca, Anamarija Fofonjka, Achyuthan N. Srikanthan, Adrien Debry, Michel C. Milinkovitch

**Affiliations:** 1 Laboratory of Artificial & Natural Evolution (LANE), Department of Genetics & Evolution, University of Geneva, Sciences III, 30, Quai Ernest-Ansermet, 1211 Geneva 4, Switzerland; 2 SIB Swiss Institute of Bioinformatics, Geneva, Switzerland; 3 Evolutionary Ecology and Biogeography Laboratory, Center for Ecological Sciences, Indian Institute of Science, Bangalore, Karnataka India

**Keywords:** Microstructure, Nanostructure, Nanograting, Snake, Scale, Phylogenetic mapping, Continuous-time Markov model, Hydrophobicity, Structural colour

## Abstract

**Background:**

Many species of snakes exhibit epidermal surface nanostructures that form complex motifs conferring self-cleaning properties, and sometimes structural iridescence, to their skin.

**Results:**

Using confocal microscopy, we show that these specialised cells can be greatly elongated along their left-right axis and that different types of nanostructures are generated by cell borders and cell surface. To characterise the complexity and diversity of these surface gratings, we analysed scanning electron microscopy images of skin sheds from 353 species spanning 19 of the 26 families of snakes and characterised the observed nanostructures with four characters. The full character matrix, as well as one representative SEM image of each of the corresponding species, is available as a MySQL relational database at https://snake-nanogratings.lanevol.org. We then performed continuous-time Markov phylogenetic mapping on the snake phylogeny, providing an evolutionary dynamical estimate for the different types of nanostructures. These analyses suggest that the presence of cell border digitations is the ancestral state for snake skin nanostructures which was subsequently and independently lost in multiple lineages. Our analyses also indicate that cell shape and cell border shape are co-dependent characters whereas we did not find correlation between a simple life habit classification and any specific nanomorphological character.

**Conclusions:**

These results, compatible with the fact that multiple types of nanostructures can generate hydrophobicity, suggest that the diversity and complexity of snake skin surface nano-morphology are dominated by phylogenetic rather than habitat-specific functional constraints. The present descriptive study opens the perspective of investigating the cellular self-organisational cytoskeletal processes controlling the patterning of different skin surface nanostructures in snakes and lizards.

**Electronic supplementary material:**

The online version of this article (10.1186/s12862-019-1411-6) contains supplementary material, which is available to authorized users.

## Background

**Fig. 1:**
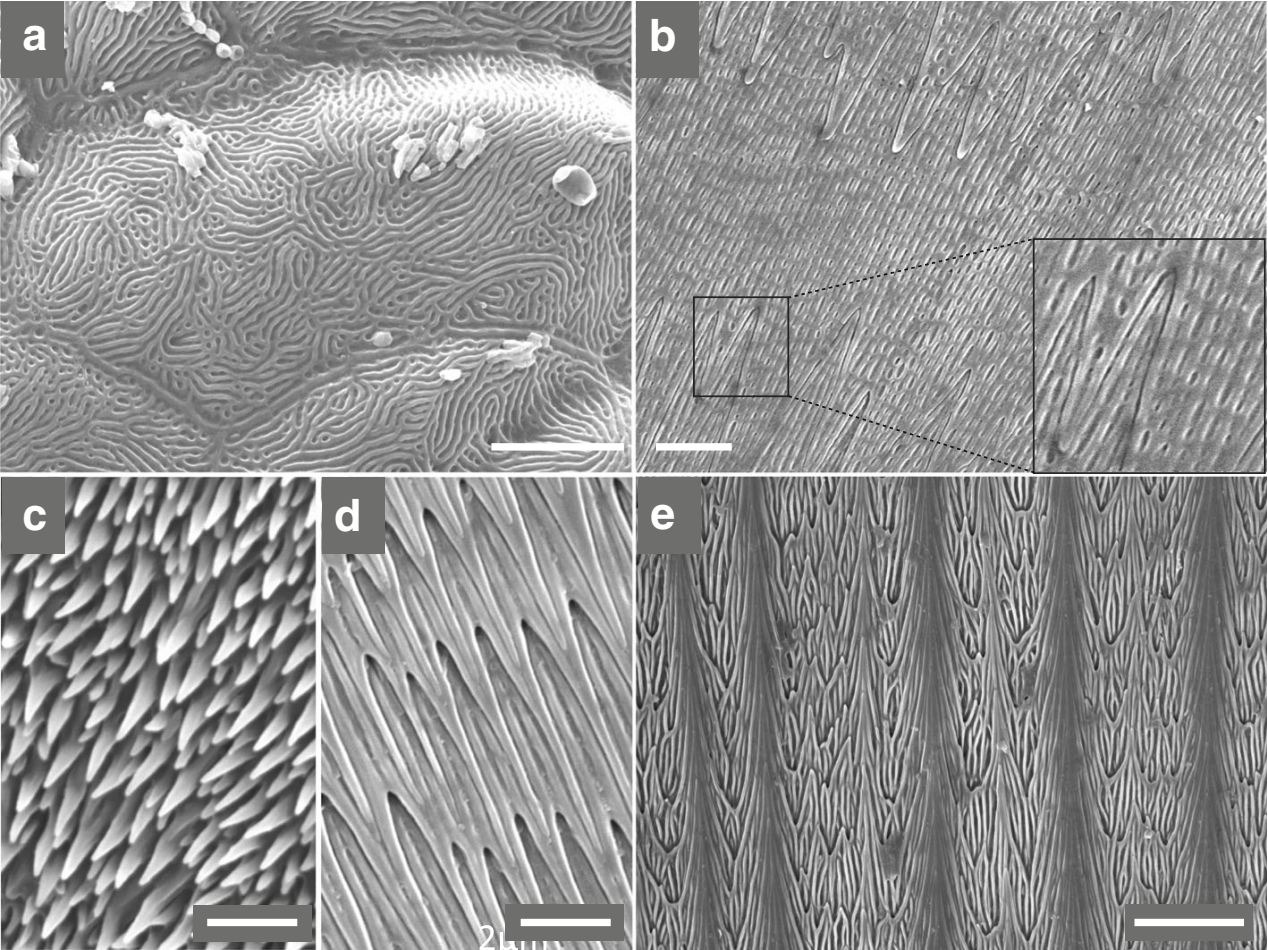
Examples of oberhautchen cell nanostructures in snakes. SEM images from sheds of **(a)** *Atropoides olmec* (Viperidae): polygonal cells with surface labyrinthine channels and regular cell borders; **(b)** *Chilabothrus strigilatus* (Boidae): wide cells with surface holes and sawteeth cell borders; **(c)** *Dendroaspis jamesoni kaimosae* (Elapidae): cells of unknown shape with a dense network of elevations; **(d)** *Boaedon fuliginosus* (Lamprophiidae): wide cells with surface channels and cell borders exhibiting long digitations; and **(e)** *Philothamnus angolensis* (Colubridae): ‘wide’ cell shape with surface covered with ‘straight channels’, and cell borders exhibiting ‘mild’ digitations; the cell surface also exhibits ‘ridges’, i.e., inverted-gutter deformations that run along the cranial-caudal axis. Scales (white bars): 5 μm (a,b), 2 μm (c,d) and 10 μm (e)

Many species of Squamates (lizards and snakes) exhibit submicron-sized surface gratings (periodic surface deformations) of their skin, i.e., at the apical surface of the *oberhautchen* cells (the outermost layer of cells in the s*tratum corneum* [1]). These sub-cellular structures, termed ‘scale microstructures’, ‘micro-dermatoglyphics’, ‘nanostructures’, or ‘nanogratings’, exhibit a variety of forms that can be broadly categorised as regular or irregular ‘digitations’, ‘holes’, and ‘channels’ [2–5]. Figure 1 illustrates some of these structures observed in snakes. This diversity of morphologies has been suggested to reflect a corresponding diversity of ecological specialisations because nanostructures can generate structural iridescence (due to light interference) in the visible range of light frequencies [6–8] and can provide their carriers with mild to extreme hydrophobicity that greatly helps keep their skin clean [9–11]. Larger elements (at spatial scales substantially larger than the micrometer) can conversely show dirt-gripping properties. For example, the body scales of some fossorial Uropeltid snakes bear regular nanoscopic depressions and digitations that confer spectacular hydrophobicity and iridescence, whereas the blunt caudal tip is covered in much larger spinules and irregular pits that cause the accumulation of dirt in the form of a protective caudal plug [12].

Nanograting morphologies not only differ among species and between body areas but also during post-natal development and within individual scales [13, 14]. Indeed, each scale exhibits a smooth gradient of nanostructure morphologies between their cranial and caudal ends, with the cranial pattern (i.e.*,* at the base of the scale) being less derived, i.e., similar to the one covering the scales of neonatal individuals.

A recent analysis [15] attempted for the first time to probe the diversity of nanostructures in snakes by described the ventral and dorsal surface nanogratings found in 41 species from the Boidae, Pythonidae, and Elapidae families. This study established characters such as cell shape, cell boundary, and cell surface morphology and enumerated hypotheses for potential links between specific structural features and ecological characters (e.g., the potential presence of longer digitations in arboreal species) but the authors did not find any conclusive correlation between scale nanomorphology and ecological characters.

Here, we provide a more extensive analysis (with a much larger number of species) of the evolution of nanogratings in snakes. First, we use scanning electron microscopy (SEM) to identify and characterise the nanograting morphologies observed in 353 species (Additional file 1: Table S1 and MySQL relational database at https://snake-nanogratings.lanevol.org) spanning 19 of the 26 families of snakes. Second, we use confocal microscopy to unambiguously identify for the first time the shape and spatial organisation of oberhautchen cells. Third, we use a continuous-time reversible Markov model for the phylogenetic mapping of all characters investigated. Fourth, using phylogenetic generalised least square regression and phylogenetic generalised linear mixed model methods, we find that some of our defined characters are co-dependent (i.e.*,* correlated), resulting in two major groups of nano-patterns: polygonal cells with regular cell borders versus elongated cells with cell border digitations. These analyses indicate that phylogeny constrains nanograting morphology whereas life habits (aquatic, terrestrial, fossorial and arboreal) do not significantly co-vary with any of the nanomorphological characters investigated, as illustrated by species of the same sub-family living in dramatically different environments but harbouring virtually the same structures. Our work aims at mapping scale nanostructures on the phylogeny of snakes, but also at guiding the identification of the developmental cellular mechanisms that generate diversity and complexity in surface nanograting structures in Squamates.

## Results

### Confocal microscopy of oberhautchen cells

**Fig. 2:**
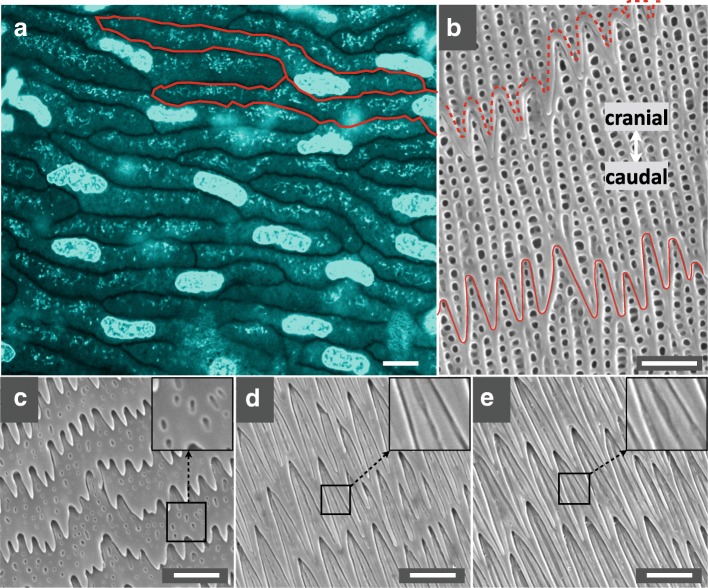
Oberhautchen cells’ morphology in *B. fuliginosus*. **(a)** Confocal microscopy image of SYTO9-labelled oberhautchen cells at the tip of an embryonic dorsal scale (day 46 post-ovoposition); the borders of two cells are highlighted in red; **(b)** SEM image of nanogratings at the tip of a scale in a neonate; the cranial and caudal borders are indicated with dashed and plain lines, respectively; **(c-e)** nanograting pattern transition (short to long digits and surface holes to surface channels) from the base **(c)** to the middle **(d)** to the tip **(e)** of an adult scale. Scale (white bars): 10 μm (a) and 2 μm (b-e)

To image the oberhautchen cells, located below the two to three thin layers of peridermal cells that cover snake embryos, we used confocal microscopy on biopsies of African House Snake (*Boaedon fuliginosus*) embryonic skin dyed with the Syto9 Green Fluorescent Nucleic Acid Stain [16] (Fig. 2a). While the nuclei appear bright, the cytoplasm is less intensely labelled, probably because of a combination of unspecific labelling and binding to cytoplasmic RNA. Figure 2a shows that the oberhautchen cells can be dramatically elongated (in comparison to a regular polygon) along the left-right axis, as suggested previously [15]. SEM images of shed skin in juveniles of the same species indicate that short digitations, formed by the caudal border of each cell, overlap the cranial side of the next cell, whereas the surface of cells exhibit chains of sharp depressions (hereafter called ‘holes’). Note that, in adults, cell borders at the base of each scale form short digitations with some holes at the cell surface (Fig. 2c) while, toward the middle (Fig. 2d) and tip (Fig. 2e) of the scale, the cell borders form longer and pointier digitations and the cell surface bears straight channels instead of holes. The shape of the cranial side of each cell is difficult to assess because it is covered by caudal digitations of the previous (cranial) cell. However, confocal analysis of an embryonic sample in which some cells are damaged (Additional file 2: Movie S1) reveals that the cranial side of each cell is similarly deformed. We therefore suggest that caudal and cranial borders of cells jointly deform but the caudal digitations of a given cell (plain line in Fig. 2b) eventually cover and conceal the cranial edge (dotted line in Fig. 2b) of the next cell. Note that we compared nanogratings in fresh skin biopsies versus shed skin and confirmed that the morphology of nanogratings is maintained in moults, greatly facilitating the collection of samples from a large number of species.

### Character assignment

**Fig. 3:**
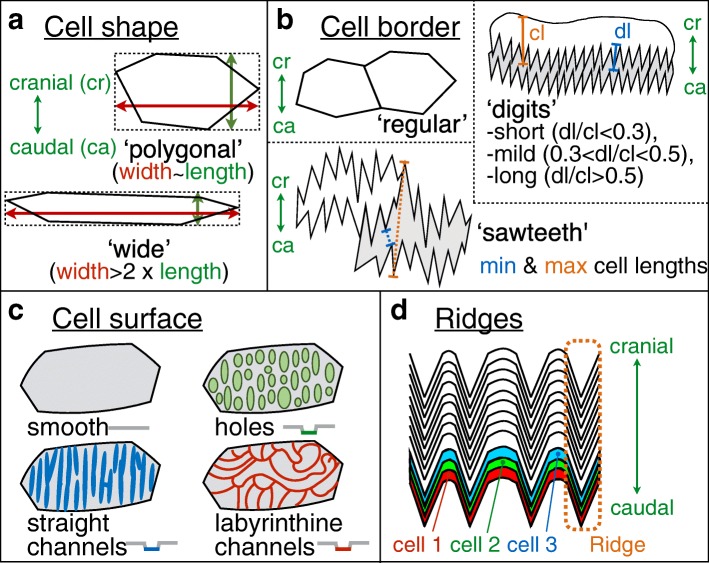
Characters used to describe and classify nanostructures. Schematic representation of the four characters used in this study: **(a)** cell shape (alternative states: ‘polygonal’ or ‘wide’); **(b)** cell border (‘regular’ or ‘digits’ or ‘sawteeth’); **(c)** cell surface (‘smooth’ or ‘holes’ or ‘straight channels’ or ‘labyrinthine channels’); and **(d)** ridges (‘present’ or ‘absent’). Abbreviations: cr, cranial; ca, caudal; dl, digit lenth; cl, cell length

**Fig. 4:**
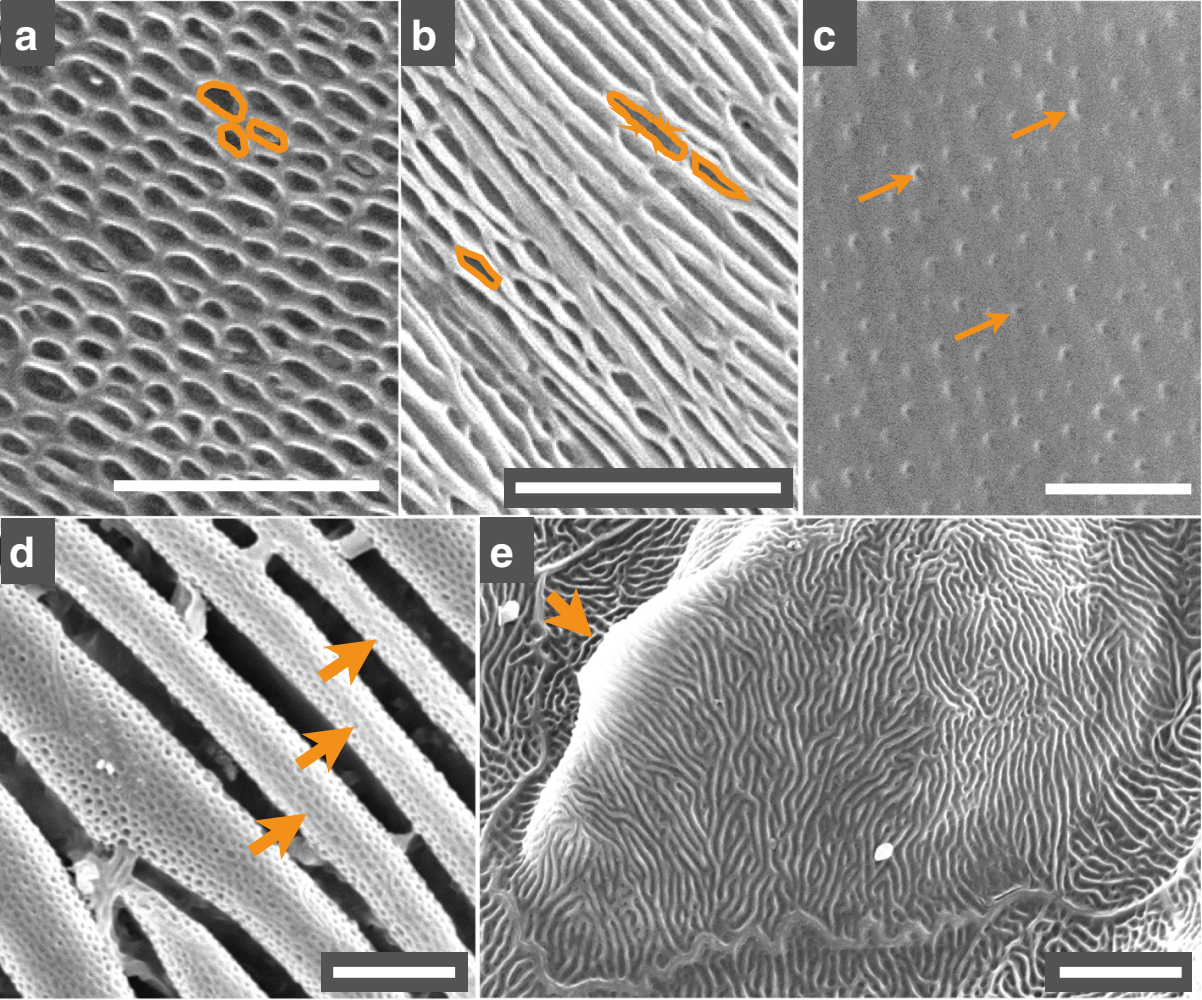
Cell surface secondary structures ignored in the analyses. The ‘cell surface’ character can take four alternative possible states: ‘smooth’, ‘holes’,‘straight channels’ or ‘labyrinthine channels’. However, **(a)** roundish and **(b)** elongated holes (some of which are highlighted in orange) were not differentiated. We also ignore: **(c)** very small cell surface pits (orange arrows), **(d)** ‘tubular’ larger structures (here, in *Pareas carinatus*), and **(e)** low-frequency geometries, such as this large elevation (orange arrow) in *Bitis gabonica*. Additional details are given in the Material and Methods section. Scales (white bars): 5 μm

To investigate the diversity of nanogratings in snakes, we performed SEM imaging on shed skin samples from 308 species of snakes and gathered published SEM results from 45 additional species (26 from [17] and 19 from [15]). These 353 species span most lineages of snakes as they represent 19 of the 26 families listed in The Reptile Database [18]. Note that we focus on the structures found at the tip of dorsal scales because they display the largest diversity of morphologies across species, due to their more developmentally-derived state. For each species, we categorised the nanostructures and associated oberhautchen cells with four morphological characters (Fig. 3): ‘cell shape’ (possible states = ‘polygonal’ or ‘wide’), ‘cell border’ (‘regular’ or ‘short/mild/long digitations’ or ‘sawteeth’), ‘cell surface’ (‘smooth’ or ‘holes’ or ‘straight channels’ or ‘labyrinthine channels’), and the absence or presence of ‘ridges’. Ambiguity was prevented by grouping similar structures (holes and chains; Fig. 4a-b), by ignoring dim structures (pits; Fig. 4c), and by ignoring secondary larger microstructures (Fig. 4d-e). When a character state could not be unambiguously scored (e.g., when cell shape or surface was not visible because of the presence of a dense network of elevation; Fig. 1c), it was classified as ‘unknown’. Details on characters and their mutually-exclusive states are given in the Materials and Methods section.

Figure 1 illustrates the characterisation of the nanostructures and oberhautchen cells in five species. *Atropoides olmec* (Viperidae, Fig. 1a) shows a ‘polygonal’ cell shape, ‘regular’ cell borders, a cell surface covered with ‘labyrinthine channels’, and ‘absent’ ridges. *Chilabothrus strigilatus* (Boidae, Fig. 1b) harbours a ‘polygonal’ cell shape (not shown), ‘sawteeth’ cell borders, a cell surface covered with ‘holes’, and ‘absent’ ridges. *Dendroaspis jamesoni kaimosae* (Elapidae, Fig. 1c) has an ‘unknown’ cell shape and ‘unknown’ cell surface (because of a dense network of elevations hides the cell borders and cell surface), unknown cell borders, and ‘absent’ ridges. Note that we did not formally identify whether the long and elevated protuberances correspond to modified cell border digitations or originate from the cell surface. *Boaedon fuliginosus* (Lamprophiidae, Fig. 1d) harbours a ‘wide’ cell shape, cell borders with ‘long digits’, a cell surface covered with ‘straight channels’, and ‘absent’ ridges. Finally, *Philothamnus angolensis* (Colubridae, Fig. 1e) possesses a ‘wide’ cell shape, ‘mild digits’ cell borders, a cell surface covered with ‘straight channels’, and ‘present’ ridges. The full character matrix with, for each of the 353 scored species, the state of each of the four morphological characters as well as a crude life habit classification is shown in Additional file 1: Table S1. The same character matrix, as well as one representative SEM image of each of the corresponding species, is available in a MySQL relational database at https://snake-nanogratings.lanevol.org.

We realise that some of the characters used in our study might not be independent. Note however that *(i)* we test below for the presence of such dependences, and *(ii)* if present, dependences among characters are not problematic in our analyses because we are not using these characters to infer phylogenies but to map characters on an established phylogeny of snakes to evaluate how these surface nanostructures evolved.

### Model of character evolution and phylogenetic signal

Inference of proper hypotheses regarding trait evolution requires that a correct model of character evolution and a correct phylogenetic tree are used [19]. Here, our analyses are conditioned upon the use of the time-calibrated tree of squamates (Additional file 3) provided by Tonini et al. [20]. To validate the character evolution model, we performed a statistical comparison of so-called ‘Mk models’ [21], i.e., Markov-chain models that describe the evolutionary change of discrete characters across a phylogenetic tree using a rate transition matrix [21, 22]. For each of the nano-morphological characters and simple life habit classification, we use a maximum likelihood (ML) estimator to fit three models differing by their rate transition matrix: *(i)* the ER model where all rates are equal, *(ii)* the SYM model where the rates from state *i* to state *j* is equal to the reciprocal rate from *j* to *i*, and *(iii)* the ARD model where all rates can assume different values. Note that we additionally compared, using Akaike information criteria, if applying Pagel’s tree transformation coefficients (λ, δ, κ [21, 22]) significantly improves each model (in terms of information discrepancy). In a tree transformation perspective, Pagel’s λ compresses internal branches to measure the dependence of trait evolution among species. Using λ = 1 leaves the tree unchanged while branches are compressed for λ < 1. When λ = 0, all branches are collapsed such that the character investigated evolves independently among species [21, 23]. Pagel’s δ coefficient manipulates the rate matrix across the phylogenetic tree: when δ < 1 or δ > 1, transition rates slow down or accelerate, respectively, from the root to the tip branches. Finally, the κ coefficient raises all branch lengths by a power κ to evaluate if the trait under investigation evolves gradually or with a punctuated dynamic [21, 22, 24]. The goal of these analyses is to identify a suitable model for character mapping on the snake phylogeny. Much additional information is provided in the Materials and Methods.

Additional file 4: Table S2 indicates that the ARD model, without application of Pagel’s tree transformation coefficients, yields the best fitting scores (based on Akaike information criteria; see Material and Methods) for the ‘cell shape’ and ‘ridges’ characters. Note that for the ‘cell shape’ character, the ARD with λ or δ coefficients are only marginally less favoured. On the other hand, for the ‘cell borders’ and ‘cell surface’ characters, the SYM model is preferred, with tree transformation parameter λ = 0.966427 (*p*-value = 3.92∙10^− 4^, compared to SYM with no transformation) and λ = 0.889429 (*p*-value = 1.97∙10^− 2^, compared to SYM with no transformation), respectively. Hence, the tree was rescaled with the corresponding value of λ prior to the mapping of the ‘cell border’ and ‘cell surface’ characters (see below). Finally, the SYM Mk model without transformation is the best fit for the life habit character (Additional file 4: Table S2).

Given that the best fit was obtained without the transformation coefficient λ for two nanomorphological characters and the life habit character, as well as with a λ value close to one for the last two characters, we can safely consider for the presence of substantial phylogenetic signal for all the characters investigated. To more formally test for the presence of such a signal, we searched (separately for each character) for the value of λ that maximises the likelihood of the tree given the character state distribution and the model. The likelihood-ratio test (H_0_: λ = 0) significantly rejects, for each character, the independence of character evolution from the phylogenetic tree (conforming the presence of phylogenetic signal): ‘cell shape’, λ = 0.98 (*p*-value = 8.63 × 10^− 32^); ‘cell border’, λ = 0.97 (*p*-value = 1.66 × 10^− 88^); ‘cell surface’, λ = 0.89 (*p*-value = 2.84 × 10^− 22^); *‘*ridges’, λ = 1 (*p*-value = 2.39 × 10^− 21^); and ‘life habit’, λ = 1.00 (*p*-value = 4.98 × 10^− 105^). The optimal value of the coefficient is very close to 1 for four of the five characters, whereas the ‘cell surface’ character presents a non-smooth likelihood profile (i.e., with oscillations), probably explaining the somewhat smaller optimal value of λ (slightly below 0.9).

### Phylogenetic mapping

**Fig. 5:**
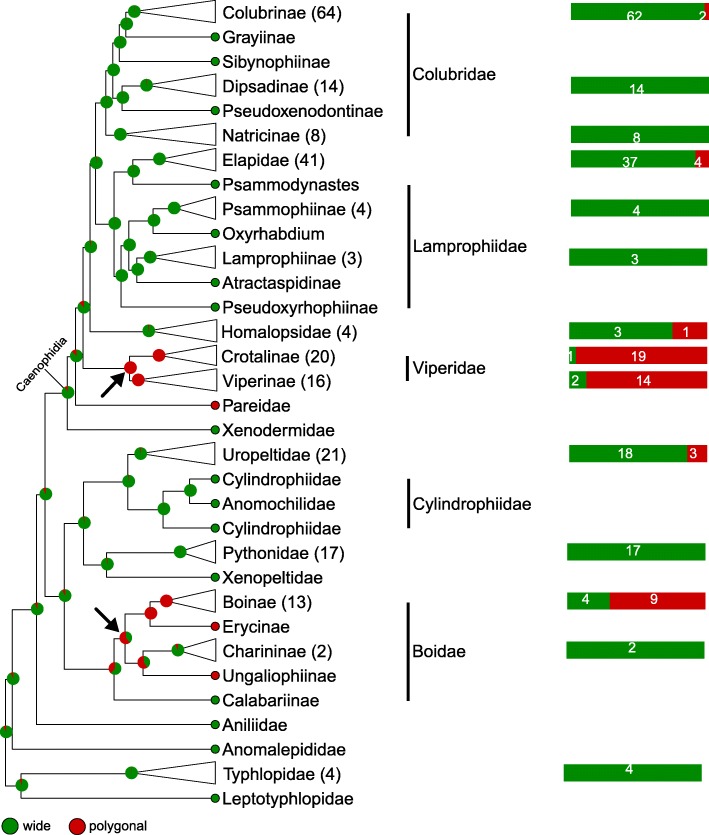
Stochastic mapping of the Cell Shape character. Green, ‘wide’; red, ‘polygonal’; plain arrows, substitution to ‘polygonal’

**Fig. 6:**
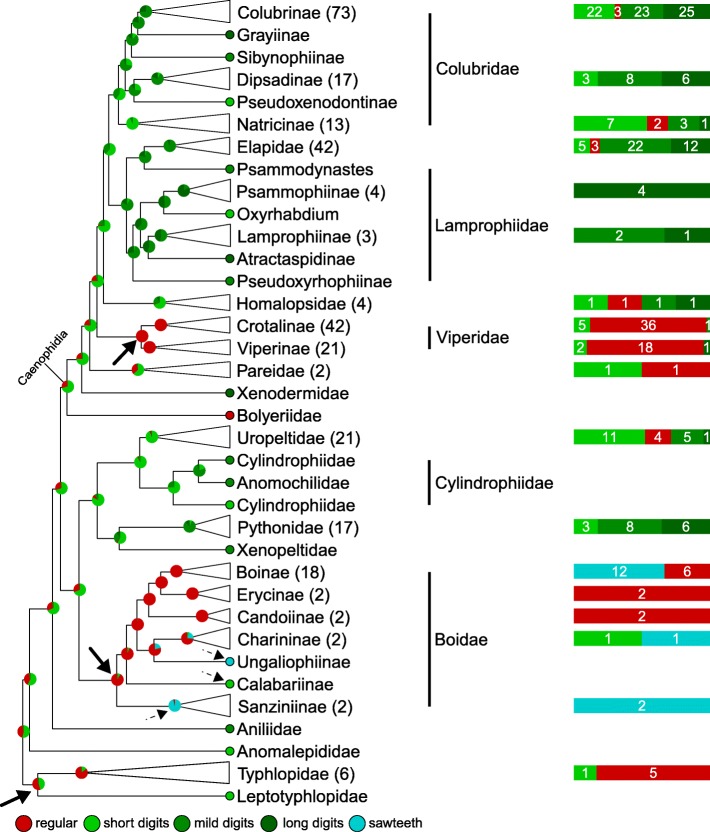
Stochastic mapping of the Cell Border character. Red, ‘regular’; light green, ‘short digits’; mild green, ‘mild digits’; dark green, ‘long digits’; blue, ‘sawteeth’; plain arrows, primary substitutions from the ancestral state; dashed arrows, secondary changes

**Fig. 7:**
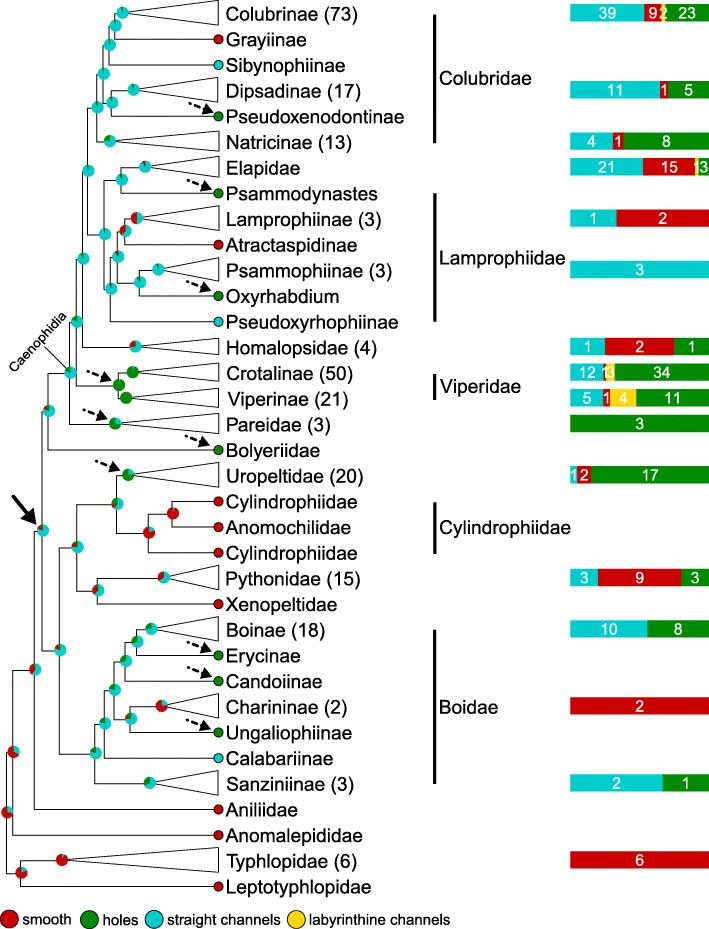
Stochastic mapping of the Cell Surface character. Red, ‘smooth’; green ‘holes’; blue, ‘straight channels’; yellow ‘labyrinthine channels’; plain arrow, possible basal transition to ‘straight channels’; dashed arrows, secondary substitutions to ‘holes’

**Fig. 8:**
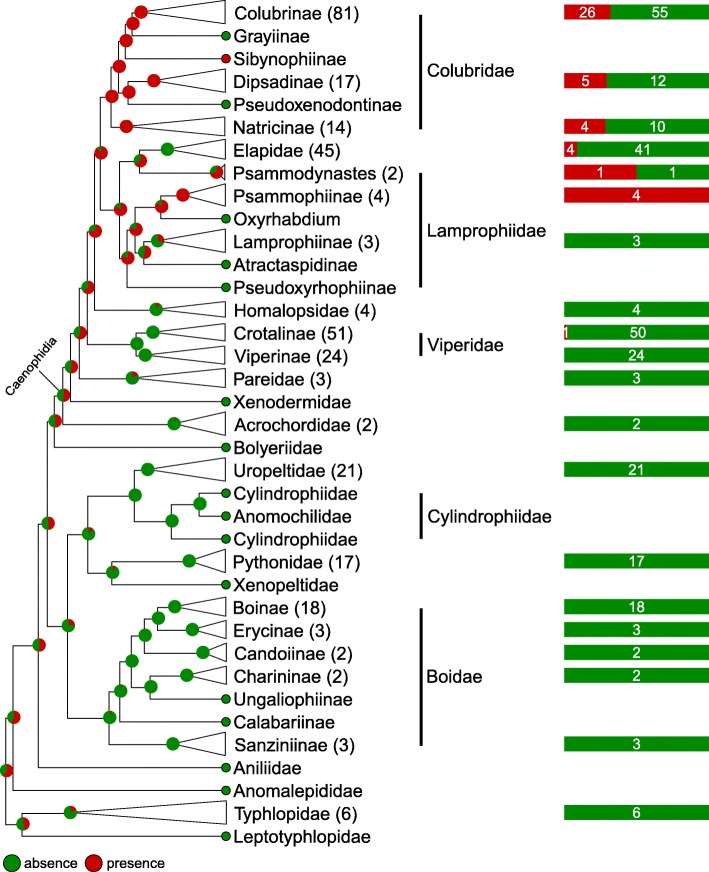
Stochastic mapping of the Ridge character. Green, ‘absence’; red, ‘presence’

**Fig. 9:**
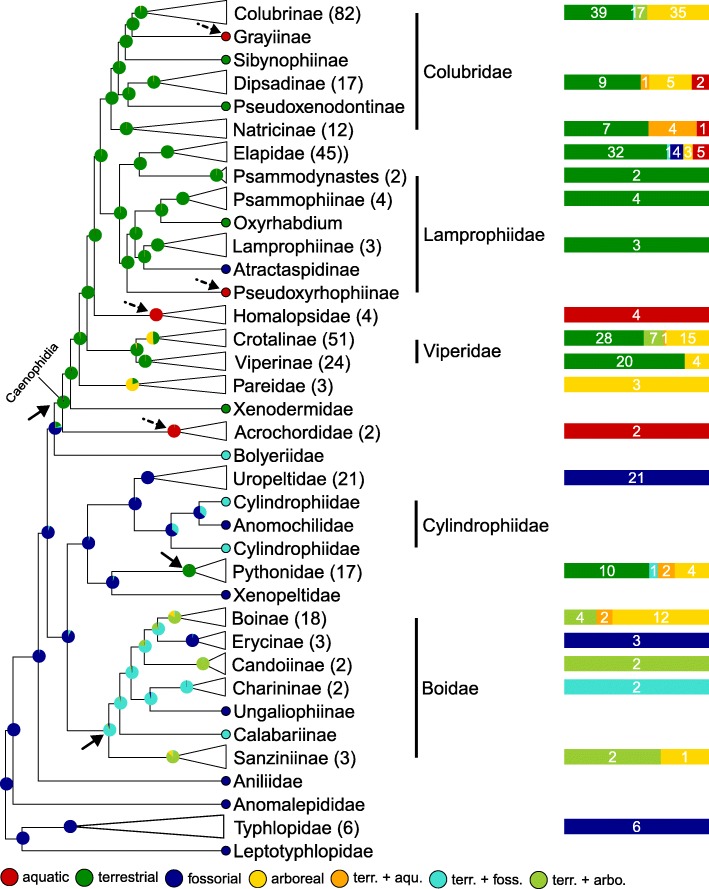
Stochastic mapping of the Life Habit character. Red, ‘aquatic’; dark green, ‘terrestrial’; blue, ‘fossorial’; yellow, ‘arboreal’; orange, ‘aquatic + terrestrial’; turquoise, ‘terrestrial + fossorial’; light green, ‘terrestrial + arboreal’. Plain arrows: convergent transitions from fossorial to terrestrial or to fossorial+terrestrial habitats; dashed arrows: secondary transitions to the aquatic environment. Multiple transitions to the arboreal state have also occurred but are not marked by arrows

**Fig. 10:**
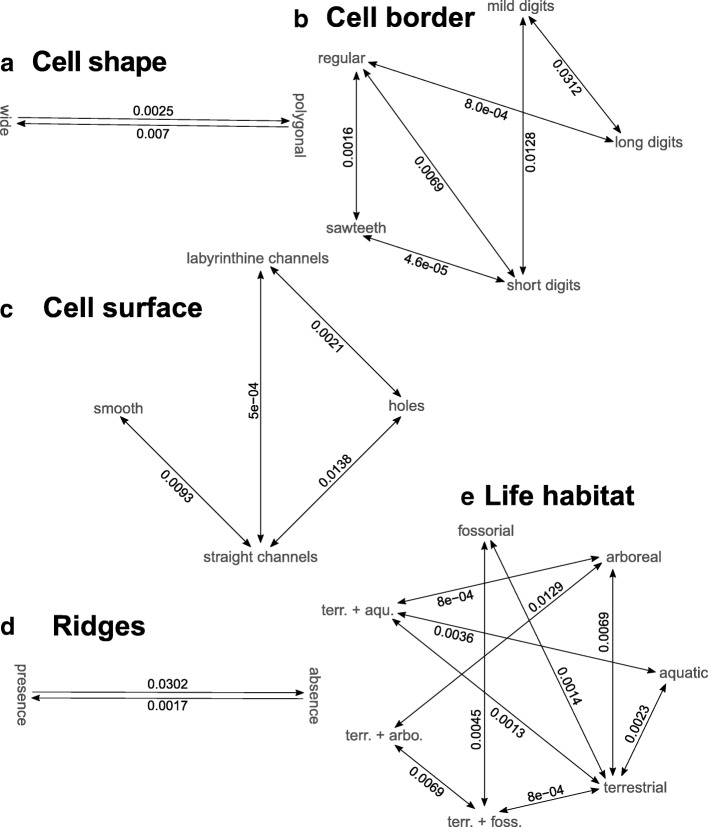
Estimated rate transition matrices for each nanomorphological character. Rates lower than 10^− 6^ are set to zero and corresponding arrows are not drawn. **(a)** Cell shape, **(b)** Cell borders, **(c)** Cell surface, **(d)** Ridge, **(e)** Life habit

Here, based on a time-calibrated tree of squamates [20] and the use of continuous-time reversible Markov models discussed above, we perform phylogenetic mapping and Bayesian ancestral state reconstruction of each of the four nanomorphological characters and one simple life habit character scored in each of the 353 snakes listed in Additional file 1: Table S1 and at https://snake-nanogratings.lanevol.org. When a character could not be scored for a species, that species was pruned from the phylogeny for the mapping of that specific character. The results are summarised in Figs. 5, 6, 7 and 8 in which clades are collapsed at the genus/sub-family or family levels when appropriate for facilitating visual inspection of phylogenetic mapping. Numbers between parentheses and colour bars next to collapsed clades indicate the number of scored species and the proportions of species exhibiting different states, respectively. Pie-charts at internal nodes indicate the posterior probability of ancestral state(s). Uncollapsed (species-level) trees are provided as Additional file 5: Figure. S1, Additional file 6: Figure. S2, Additional file 7: Figure. S3, Additional file 8: Figure. S4, Additional file 9: Figure. S5, Additional file 10: Figure. S6, Additional file 11: Figure. S7, Additional file 12: Figure. S8, Additional file 13: Figure. S9, Additional file 14: Figure. S10, Additional file 15: Figure. S11. Additionally to nanomorphological characters, the ecological history of snakes is mapped in Fig. 9 using a very simple classification (aquatic, terrestrial, fossorial and arboreal). These analyses provide an estimate of how nanomorphological character states or life habits changed across the phylogeny and, hence, how the distribution of character diversity and ecological history in extant snakes was achieved. In parallel, based on the best fitted ML model, we estimate the transition rate matrix for each of the characters (Fig. 10) to obtain a more quantitative description of state transitions across the phylogeny of snakes; note that we consider all rates with a value < 10^− 6^ to be zero. Results are described and discussed in more details below for each character separately.

#### Cell shape (Fig. 5 and Additional file 5: Figure. S1)

Clearly, the ‘wide’ state (oberhautchen cells more than twice wider than long) is prevalent in snakes whereas the ‘polygonal’ state is dominant only in vipers (family Viperidae) and in three sub-families (Boinae, Erycinae and Ungaliophiinae) of boas whereas it also appears, albeit at low frequencies, in Colubrinae, Elapidae, Homalopsidae, and Uropeltidae. Note that our single representative of Pareidae also exhibits polygonal oberhautchen cells; additional samples are required to infer the character states frequencies in this small family of about 20 species. An important result of this mapping is that the cell shape state distribution across the phylogeny of snakes necessarily requires convergent substitutions. The most probable scenario involves two changes in deep nodes from ‘wide’ to ‘polygonal’ (black arrows in Fig. 5): once at the basal node of Viperidae and once within Boidae. Clearly, when considering the species-level phylogeny, additional homoplastic events occurred at shallower nodes: inferential mapping suggests a total of ten changes from ‘wide’ to ‘polygonal’, and six reversals from ‘polygonal’ to ‘wide’ (Additional file 5: Figure. S1). As there are many more species with the ‘wide’ state than the ‘polygonal’ state, these results suggest that the ‘polygonal’ to ‘wide’ reversal is easier than the ‘wide’ to ‘polygonal’ transition. This is supported by the estimated transition rate matrix (Fig. 10a) that shows a nearly three times faster rate from ‘polygonal’ to ‘wide’ than for the converse transition.

#### Cell borders (Fig. 6 and Additional file 6: Figure. S2)

In addition to the ‘sawteeth’ and ‘regular’ states, this character can present ‘digitations’ that we separated into three sub-states (‘short’, ‘mild’, and ‘long’ digitations; see Materials and Methods). For the sake of simplicity, we will discuss the results pertaining to the cell border character with the three types of digitations grouped. The most probable ancestral state for cell borders in snakes is the presence of ‘digitations’, (although ‘short digits’ present only a slightly higher posterior probability than ‘regular’ cell borders) followed by various transitions to other states. The three deepest transitions (plain black arrows in Fig. 6) are ‘digitations’ to ‘regular’ in the ancestor of Typhlopidae and Leptotyphlopidae, as well as at the base of the Boidae, and Viperidae families. Note that multiple transitions to ‘sawteeth’ and one reversal to ‘digitations’ occurred in subclades of Boidae (e.g., dashed black arrows in Fig. 6). Similarly, multiple reversals occurred within the Viperidae family from the ‘regular’ to the ‘digitations’ state (Additional file 6: Figure. S2). It is worth noting that the digitations observed in species belonging to the Caenophidia exhibit sharp tips (Additional file 7: Figure. S3a) whereas those observed in all other taxa (Uropeltidae, Cylindrophiidae, Anomochilidae, Pythonidae, Xenopeltidae, and Typhlopidae) are characterised by rounded tips (Additional file 7: Figure. S3b). This observation suggests that the presence of digitations is beneficial to their bearers, independently of the exact shape of these nanostructures. The transition matrix among cell border character states (Fig. 10b) indicates obvious transition constraints. First, the ‘regular’ state can only change to ‘short’ or ‘long’ digitations (with a much higher rate for the former) or to the ‘sawteeth’ state, while ‘sawteeth’ can convert additionally to ‘short’ digits. In addition, ‘short’ digits cannot evolve to ‘long’ ones without transitioning first to the ‘mild digits’ state.

#### Cell surface (Fig. 7 and Additional file 8: Figure. S4)

Because the four successive basal snake families (Leptotyphlopidae, Typhlopidae, Anomalepididae, and Aniliidae) exhibit ‘smooth’ oberhautchen cells, the mapping indicates this character state as ancestral for all snakes. On the other hand, the presence of ‘straight channels’ is strongly supported as the ancestral state for the clade including all remaining snake families (black plain arrow in Fig. 7), followed by reversals to ‘smooth’ in multiple shallower lineages. Statistical mapping of this character also indicates that ten independent switches to ‘holes’ are likely to have occurred (dashed black arrows in Fig. 7) in the Colubridae, the Lamprophiidae, the Viperidae, the Pareidae, the Boyeriidae, the Uropeltidae and in some sub-families of Boidae. It is also noteworthy that substitutions to ‘labyrinthine channels’ occurred multiple times but in much shallower branches within the Caenophidia clade (Additional file 8: Figure. S4): seven times in the Viperidae, once in the Elapidae, and twice in the Colubridae. The combination of high homoplasy and low frequency of the ‘labyrinthine channels’ state might indicate its relatively low developmental robustness, possibly because the ranges of the parameters values generating that self-organisational steady state define a small hyper-volume within the otherwise large phase space.

The distribution of shapes of the structures classified as ‘holes’ and ‘straight channels’ (Additional file 9: Figure. S5a) suggests the existence of a continuum between these two states and that channels simply evolved through the elongation of holes. This is supported by the inferred rate matrix of the discrete character model (Fig. 10c) where the ‘straight channels’ state can virtually only be reached via the ‘holes’ state. Although the classification of a depression as a ‘hole’ or a ‘straight channel’ is based on arbitrary (but objective) geometrical criteria (see Materials and Methods), the majority of species with these states harbour only ‘holes’ or only ‘straight channels’, rarely a mix of both (Additional file 10: Figure. S6). This observation supports the idea that, although the shape’s distribution is continuous, these states are distinct and can quickly switch from one to the other.

While the oberhautchen cell surfaces in Elapidae are nearly exclusively ‘smooth’ or covered in ‘straight channels’, it is worth noting that all Caenophidia species harbouring ‘smooth’ cell surfaces (marked by stars in Additional file 8: Figure. S4) actually possess very small depressions that do not pass the algorithm’s requirements to be classified as ‘holes’ (e.g., Fig. 4c, < 3% of image surface covered). Only the species of the three basal families of snakes (Leptotyphlopidae, Typhlopidae, and Anomalepididae) appear to have truly smooth cell surfaces (i.e., devoid of any dimple). Finally, note that ‘holes’ and ‘labyrinthine channels’ cannot be unambiguously differentiated in some species (Additional file 11: Figure. S7), possibly affecting the inference of correct rates between these states (Fig. 10c).

#### Ridges

Bayesian mapping (Fig. 8 and Additional file 12: Figure. S8) suggests that ‘presence’ and ‘absence’ of ridges are two states with similar posterior probabilities at the most ancestral nodes. This inference is non-intuitive in the maximum parsimony framework because basal lineages contain species exclusively without ridges. However, this result is consistent with the stochastic mapping framework: Bayesian inference indicates that the gain of ridges occurred several times within Caenophidia, followed by even more numerous reversals to ‘absence of ridges’. This explains that the corresponding inferred rate of losses is about 18 times higher than that of gains (Fig. 10d). This high level of homoplasy, combined with the large branch lengths of basal lineages, explains that ‘presence’ and ‘absence’ of ridges exhibit similar posterior probabilities at the most ancestral nodes.

Although some ex-Caenophidia families (e.g., Uropeltidae and Pythonidae) also bare ‘digitations’ (Fig. 6, Additional file 6: Figure. S2), ridges are found exclusively in families that harbour ‘sharp digits’ (Additional file 7: Figure. S3a), suggesting that the evolution of ‘ridges’ was favoured by the alignment and overlap of sharp digits.

#### Life habit (Fig. 9 and Additional file 13: Figure. S9)

Using a very simple life habit classification (aquatic, terrestrial, fossorial, and arboreal), we mapped this ecological character on the phylogeny of snakes. Note that because some species cannot be strictly categorised using this simplistic scheme, we additionally considered the following three combinations as distinct states: terrestrial+aquatic, terrestrial+fossorial, and terrestrial+arboreal. Figure 9 shows that ‘fossorial’ is, by far, the most likely state for the ancestor of all snakes, in agreement with recent phylogenomic and morphological studies [25, 26]. Convergent transitions from the fossorial to the terrestrial life habit (plain arrows in Fig. 9) most likely occurred at the base of Caenophidia, at the base of Pythonidae and at the base of Boidae. This was likely followed by multiple transitions to aquatic (dashed arrows in Fig. 9: Grayiinae, Pseudoxyrhophiinae, Homalopsidae, Acrochordidae) and to arboreal life habits (e.g., many Boidae and Colubridae). The transition rates matrix inferred from our analyses (Fig. 10e) indicates that ‘terrestrial’ is the only state accessible from all other states (except for the ‘terrestrial+arboreal’ state that is accessible only via ‘terrestrial+fossorial’ or ‘arboreal’ life habit), suggesting that transitions among most of the other states require an intermediate terrestrial state.

#### Correlations among characters

To investigate potential developmental or functional links among nano-morphological characters, we tested for the presence of correlations among the four oberhautchen cell characters and the simple life habit classification. Given the lack of consensus on which method to apply for discrete characters [24], we used both the phylogenetic generalised least squares regression (PGLS) approach [27] and the phylogenetic generalised linear mixed model (PGLMM) method [28] (see details in the Material and Methods). PGLS yields simple linear coefficients and deviations of the mean value of the variable response (considered here as pseudo-continuous), while PGLMM quantifies the influence of the probability (in log-odds ratio) of one state of a character as a function of a state of another character. Although the PGLMM approach allowed overall good convergence of the MCMC chain, it yielded poor significance levels of the coefficients in most cases, probably because of lack of knowledge on the prior probabilities [24] (here, we use the same prior for the variance of all characters).

Nevertheless, both the PGLS and PGLMM methods indicate that the ‘cell border’ and ‘cell shape’ characters are correlated (Table 1; Additional files 16 and 17): PGLS yields global and coefficient *p*-values of 7.2 × 10^− 3^ and PGLMM yields coefficients *p*-values < 2.7 × 10^− 2^ (while it does not provide a global model *p*-value). This result defines two groups of cells types, ‘polygonal and regular’ versus ‘wide with digits’, and the covariation is easily noticed in the form of simultaneous transitions of cell shape and cell borders in both the Boidae and Viperidae families. Indeed, although most other families exhibit both ‘wide’ cells and ‘digits’, Boidae and Viperidae show ‘polygonal’ cells (Fig. 5) and either ‘regular’ or ‘sawteeth’ cell borders (Fig. 6). The co-occurrence of substitutions to these states is also observed in the *Melanophidium* genus of the Uropeltidae family (black circle arc in Additional file 5: Figure. S1 and Additional file 6: Figure. S2). Such parallel covariation in separate lineages suggests the presence of developmental constraints making these characters non-independent.

**Table 1:** Correlations among characters: Summary of PGLS and PGLMM statistics

Response Variable	Predictor Variable	PGLS	PGLMM
Cell Border			
	Cell Shape	Significant	Significant
	Cell Surface	Non Significant	Significant
	Ridges	Significant	Significant
	Life Habitat	Non Significant	Non Significant
Cell Shape			
	Cell Surface	Non Significant	Significant
	Ridges	Non Significant	Non Significant
	Life Habitat	Significant	Non Significant
Cell Surface			
	Ridges	Non Significant	Non Significant
	Life Habitat	Non Significant	Significant
Ridges			
	Life Habitat	Non Significant	Non Significant

The ‘cell border’ and ‘ridges’ characters are also clearly correlated (Table 1; Additional files 16 and 17): PGLS yields global and coefficient *p*-values 1.9 × 10^− 2^ and PGLMM gives one coefficient *p*-value of 4.2 × 10^− 2^, supporting our observation that ridges are exclusively found in families that harbour ‘digits’ (Figs. 6 and 8). Finally, our analyses did not uncover the presence of a correlation (Table 1; Additional files 16 and 17) between the simple life habit classification and any nanomorphological character. This result suggests that the distribution of the nanostructure diversity and complexity on snake oberhautchen cells is dominated by phylogenetic, and probably developmental, rather than functional, constrains.

## Discussion

The exact function(s) of oberhautchen cells nanogratings is (are) not fully characterised although it is very likely that they are involved in providing snakes (and some other species of squamates) with skin self-cleaning properties [29]. Indeed, nanostructures are known to substantially increase surface hydrophobicity [9–11], a property that prevents most of the organic and inorganic material found in soil and vegetation to stick to the skin. Note that all of the nanostructures (e.g., holes, channels of various forms, digits, ridges) found on the skin of snakes are likely to provide some level of hydrophobicity. Hence, this could explain the lack of correlation between the type of oberhautchen cell nanostructures and the habitat of the corresponding species: as long as they generate hydrophobicity, the exact morphology of these nanostructures is irrelevant to fitness. For example, the presence of holes on the surface versus digits at the borders of oberhautchen cells in, respectively, the Jamaican Boa (*Chilabothrus subflavus*) and the corn snake (*Pantherophis guttatus*), each generates a substantial hydrophobic skin. Therefore, developmental mechanisms generating different nanostructures at the surface of the skin of different species might be somewhat equivalent in terms of adaptive value. Of course, this interpretation should be taken with caution as habitat and ecology includes far more nuance than we have captured with our simple characterisation of habitat (terrestrial, fossorial, arboreal, aquatic); i.e., different types of nanostructures might reflect adaptations we have not identified.

Note that some species (*Xenopeltis unicolor* exhibiting digits on cell borders, *Boiga multomaculata* exhibiting holes on cell surface) are characterised by particularly organised nanostructures, i.e., holes or digits (and digit rows) are highly regular in their spatial distributions (Additional file 14: Figure. S10). Whereas it is unknown if regularly-spaced nanostructures provide higher hydrophobicity than irregular ones, it is clear that the former generate structural iridescence (due to light interference) if the length scale of the distribution period is similar to visible light wavelengths [6–8]. Hence, it is conceivable that nanostructural traits are associated to intra- or inter-specific communication and/or sexual selection rather than to habitat types. A functional analysis, characterising the optical properties, level of hydrophobicity, and friction-modifying characteristics (with different types of substrates) of each combination of nanostructures found in the major lineages of snakes might provide answers to these questions.

## Conclusions

Here, we use scanning electron microscopy to identify and characterise the diversity of oberhautchen cells nanogratings found across 353 species representing 19 of the 26 families of snakes (and 32% of all snake genera), i.e., spanning most of the Serpentes infra-order major lineages. We then use a continuous-time reversible Markov model to perform phylogenetic mapping and investigate how this diversity of morphologies was brought about.

These results suggest that the diversity and complexity of snake skin surface nano-morphology are dominated by phylogenetic, rather than habitat-specific, constraints. Obviously, this does not necessarily mean that there was no environmental effect on the evolution of the diversity observed and much additional, more detailed, ecological analyses are warranted.

Finally, a key question concerns the identification of the molecular developmental mechanisms generating these spectacular nanostructures. What are the cytoskeletal elements involved in the development of ‘holes’ and ‘digitations’? What are the self-organisational processes controlling the spatial organisation of these structures? It is likely that the development of the corn snake as a new model species [30–32] in squamates and the recent availability of its full genome sequence and reptilian transcriptome data [33, 34] will facilitate the proper investigation of these questions.

## Methods

### Specimens

Sheds from 308 species of snakes were collected from museums, vivariums, private breeders and reptile shops. These samples were used for Scanning Electron Microscopy (SEM) imaging. The SEM images of 45 additional species from previous publications [15, 17] were also used. The 353 species scored for the present analysis represent around 32% of all snake genera (169 over 522 genera [18]) and 19 of the 26 snake families. Confocal microscopy was performed on skin biopsies obtained from African House Snake (*Boaedon fuliginosus*) embryos (day post-ovoposition 46) in the colony housed in Milinkovitch’s laboratory, University of Geneva, Switzerland. Maintenance of, and experiments on snakes were approved by the Geneva Canton ethical regulation authority (authorisations GE/82/14 and GE/73/16) and performed according to Swiss law. These guidelines meet international standards.

### Confocal microscopy

African house snake skin samples were laid flat face up on a Millipore nitrocellulose membrane (0.8mu AABP) to prevent curling and placed in an Eppendorf tube. Then, 250 μl of Gibco Dulbecco’s phosphate-buffered saline (+ CaCl2 and MgCl2) + 1 μl of 1000x Syto9 Green Fluorescent Nucleic Acid Stain (ThermoFisher Scientific S34854) [16] were added and the samples were incubated 1 h at 30 °C and 300 rpm in an Eppendorf Thermomix agitator. The skin was then peeled from the filter and mounted between a microscope slide and coverslip, which was glued on to prevent detachment in the inverted confocal microscope. As the Syto9 Green dye binds to nucleic acids, the nuclei appear very bright whereas the cytoplasm is less intensely labelled, allowing the visualisation of both the nuclei and the borders of the cells with high resolution. A Zeiss LSM 780 inverted microscope was used with a Zeiss Planapo 63X Oil 1.4NA lens and 1.518 refractive index oil. The Argon 488 nm excitation laser was used and the detector frame was set between 490 nm and 600 nm to capture the entire Syto9 emission spectrum.

### Scanning Electron microscopy and character description

A dorsal sample from each moult was excised using a scalpel blade and placed on a cylindrical specimen mount covered with a carbon adhesive tab (Electron Microscopy Sciences). The samples were then coated with gold and SEM was performed with a Jeol JSM-6510LV microscope using a beam size of 40 nm and a voltage of 10 kV. Pictures were taken at 3000x and 9000x magnifications at the cranial tip, in the middle, and at the caudal tip of a scale from each sample. When the cells were bigger than the image frame at 3000x, a new image was taken at 1000x at the tip of the scale to allow visualisation of cell shape.

We defined four nanomorphological characters, each with a number of mutually exclusive states (Fig. 3). Each of the 353 studied species was categorised using the nanostructures found at the tip of the scales, which are the most developmentally-derived [14]. The corresponding state matrix is provided in Additional file 1: Table S1 as well as in a MySQL relational database at https://snake-nanogratings.lanevol.org. Note that the latter also provides one representative SEM image of each of the corresponding species.

The characters and their states are (Fig. 3): ‘cell shape’ (‘wide’ = 0 or ‘polygonal’ = 1), ‘cell border’ (‘regular’ = 0;‘short digits’ = 1;‘mild digits’ = 2;‘long digits’ = 3 or ‘sawteeth’ = 4), ‘cell surface’ (‘smooth’ = 0; ‘holes’ = 1; ‘straight channels’ = 2 or ‘labyrinthine channels’ = 3), and ‘ridge’ (‘absence’ = 0 or ‘presence’ = 1). Ambiguity was prevented by grouping similar structures (holes and chains), by ignoring dim structures (pits, Fig. 4c), and by ignoring substantially larger microstructures (Fig. 4d,e). When a character’s state was unclear (e.g., cell shape or cell surface not visible), it was classified as ‘unknown’.

For the ‘cell shape’ to be categorised as ‘polygonal’, the ratio between the width (left-right axis) and length (cranial-caudal axis) of the cell must be ≤2 (Fig. 3a). Conversely, when the cell is more than twice wider than long, it is classified as ‘wide’. When a cell’s length varies a lot, as in ‘sawteeth’ cell border, an average is taken between the longest and shortest lengths (Fig. 3b).

For the ‘cell border’ character (Fig. 3b), the states ‘regular’ and ‘sawteeth’ are easily recognised while ‘digitations’ can show various lengths. The length of digits and the total cell length were measured in ImageJ for 10 cells and averaged. The digit length was normalised by the cell length. The states of digitations were defined as ‘short’, ‘mild’, and ‘long’ when the ratio of digit length versus cell length was < 0.3, between 0.3 and 0.5, and > 0.5, respectively. Note that the absolute size ranges of short, mild and long digitations overlap as they are 0.4–4.7 μm, 0.8-5 μm, and 1.2–5.5 μm, respectively. Digitations and sawteeth are easily differentiated because the former are small (< 5.5 μm), regular, cover the cranial border of the next cell, and are specifically oriented toward the caudal end of the scale. On the other hand, sawteeth are larger (5-10 μm), irregular, and the borders of adjacent cells imbricate with each other. Note that it is sometimes difficult to discern the cell’s borders as the previous clear layer’s imprint can leave marks on the current oberhautchen cell layer. Some species exhibit a dense network of ‘elevations’ (Fig. 1c) that could either be elevated border digitations or originate from the cell surface.

The ‘cell surface’ states were defined using functionalities from the OpenCV computer vision library [35]: all contours (i.e., closed curves connecting sharp changes in pixel intensity) in each SEM image were detected. First, we applied *Contrast Limited Adaptive Histogram Equalisation* [36] to account for inhomogeneity of contrasts across the image while preventing the over-amplification of noise (Additional file 15: Figure. S11a-b). Next, a local thresholding method was applied as follows: images were divided into small tiles (100 × 100 px) and a k-means clustering method (k = 3) was applied to group the pixels intensities of each tile independently (Additional file 15: Figure. S11c). The black and white thresholding for each tile was then set to the average of the cluster centres with the lowest and middle intensities to differentiate the darkest pixels from those in the two other clusters (Additional file 15: Figure. S11d). Artefacts at the tile borders were removed by applying Gaussian filtering on the threshold values. Then image intensities were inverted and contours were detected. The contours were then classified into shapes based on the ASR criterion: A is the area of the contour (normalised by image area), S is its ‘solidity’ (i.e., the ratio of the contour surface and the surface of its bounding convex polygon), and R (≥ 1) is the ratio of the minimum bounding rectangle sides. A contour was classified as a ‘hole’ if S > 0.8, R < 6 and A is less than two standard deviations (SD) away from the mean area of detected holes on that image (blue contours in Additional file 15: Figure. S11e). This resulted in the grouping of similar structures that could be qualified as roundish and elongated holes (Fig. 4a-b). For a contour to be sorted as a ‘straight channel’ it required S > 0.5 and R ≥ 6, but no constraint on the area (A) as channels can be very long (red contours in Additional file 15: Figure. S11e). The value of R = 6 separating the two classes was selected arbitrarily as the distribution for the bounding rectangle length/width ratio is continuous and strongly decreasing (Additional file 9: Figure. S5a). The classification was improved using a training set consisting of contours (from manually-selected images) clearly belonging to a certain class. Contours that were not labeled as a ‘hole’ or a ‘straight channel’ based on the ASR criterion were still classified if a similar shape (calculated using Hu moments [37] implemented inside OpenCV *‘Match Shapes’* procedure) was found in the training set. To identify ‘labyrinthine channels’, we recognised all contours that could not be classified into ‘holes’ or ‘straight channels’ but could be subdivided into multiple straight channels based on their convexity defects (green contours in Additional file 15: Figure. S11e). To avoid miss-classification due to image quality or cell border detection, all images were manually corrected by deleting, redrawing and automatically sorting or subdividing already existing contours. Finally, we made sure to subdivide all ‘labyrinthine channels’ into ‘straight channels’ to facilitate the process of image classification (Additional file 15: Figure. S11f). At the end of the procedure, the percentage of surface covered by each type of shapes on a SEM image was calculated and the final image state was decided using a simple decision tree. If the total area of every shape was less than 3% of the total surface of the image, the state was set to ‘smooth’. Otherwise, we set the state to ‘holes’ if they had the largest covering surface. If ‘channels’ had the largest covering area, we measured their orientation and classified the image as ‘labyrinthine channels’ if the angle SD was > 25°, or as ‘straight channels’ otherwise. Given that the standard deviation of channel orientation (Additional file 9: Figure. S5b) presents a gap between 22° and 26°, we can objectively set a threshold at 25° for separating ‘straight channels’ (R ≥ 6) from ‘labyrinthine channels’ (channel orientation angles with SD > 25°). Despite these criteria, some species exhibited structures that could not be unambiguously classified as ‘holes’ or ‘labyrinthine channels’ (Additional file 11: Figure. S7).

The ‘ridge’ character refers to inverted-gutter deformations of the cell surface that run along the cranial-caudal axis (Fig. 3d).

### Phylogeny

First, we pruned the time-calibrated tree among 9754 species of squamates [20] to keep the 353 species for which we have obtained nanomorphological data. Note however that some of these species are represented by skin samples from multiple subspecies. Given that no subspecies information is provided in [20], we selected only one subspecies using the two following successive criteria: (1) if some, but not all, subspecies within a species could not be scored for one or several characters, remove the corresponding subspecies, and (2) if the different subspecies in a species exhibit different character state(s), keep the subspecies with the state(s) most frequently exhibited by the most closely-related other species. When more than one subspecies per species remained, we arbitrarily kept only one of them. This procedure removed 13 subspecies from the dataset prior to character mapping and subsequent analyses. The corresponding Newick tree file among 340 species is provided as a Additional file 3(Suppl_File_3_Tonini_SnakesCommon.nwk). As the pruning process did not remove all of the polytomies present in the original time-calibrated tree [20], and some of our analyses (e.g.*,* models of character evolution) require a dichotomous tree, we resolved these ambiguous nodes with the function *multi2di* of the *ape* package [38] of the program R [39] while imposing the length of the newly-generated branches to an arbitrarily small value (10^− 6^ of the total height of the tree); this avoids problems generated by zero branch lengths in comparative analyses functions. Note that the reverse transformation can easily be performed with the *di2multi* function of the *ape* package [38]. The 353 snake (sub) species in our dataset are listed in Additional file 1: Table S1 and in the MySQL relational database at https://snake-nanogratings.lanevol.org; in both cases, the 13 subspecies not used for phylogenetic mapping are indicated with an asterisk.

### Models of character evolution

Without a priori information about the possible character evolutionary model, we fitted and compared different rate transition matrices of the Mk model with or without Pagel’s tree transformation coefficients λ, δ, and κ [21, 22]. The Mk model for discrete characters is based on the assumptions that *(i)* the trait value can evolve through a Markov process, i.e.*,* the probability of character substitution depends only on the current state and not on previous states, and *(ii)* every state of a particular trait is equally likely to change to any other states. This model is the analogue to a Brownian motion (BM) model for continuous traits as it supposes that evolutionary changes are independent across lineages and that the rate of change is constant over time and along all branches [21, 22, 24]. We focused on three forms of the rate matrix: equal rates (ER) for all substitutions, different but time-reversible (i.e., symmetric) rates (SYM), or with all rates being different (ARD).

When using continuous characters, coefficients can be used to transform the variance-covariance matrix and fit the model to the corresponding tree. On the other hand, when using discrete characters, one cannot define a covariance matrix, such that coefficients can only be applied by transforming the tree while keeping a constant-rate Mk model [21–24]. First, the λ coefficient compresses internal branches (without affecting tip branches) to measure the dependence of trait evolution among species. A value of λ = 1 leaves the tree unchanged, i.e., the model is assumed to maximally fit the original tree. On the other hand, a λ = 0 (making the tree a star-like phylogeny) indicates independence of evolution among species. Hence, comparing the fit of the model with various values of λ provides a good approach to test for the presence of ‘phylogenetic signal’ (see next subsection). Second, the coefficient δ constitutes a quantitative measure of how rates of substitution change across the tree (i.e., through time). If δ < 1, the evolutionary rate slows down through time, while δ > 1 indicates an acceleration of the rate. In terms of phylogenetic transformation, it affects the tree by modifying nodes’ heights. Third, the coefficient κ raises the branches length by a power κ. When κ = 1, the tree is left unchanged while all branch lengths are one when κ = 0. The parameter κ can be interpreted as character changes being more or less concentrated at speciation events, i.e., it tests if evolution of a trait was punctuated or gradual [21, 22, 26].

For each character, we fitted all combinations of models (ER, SYM, ARD) and coefficients (none, λ, δ, and κ) with the function *fitDiscrete* of the *geiger* package [40] using an internal optimisation procedure supported by the functions *optim* and *subplex* (i.e., general-purpose optimisation functions provided in R [39]). We used 10^4^ starting points during the optimisation procedure and performed comparisons among all fitted models using the sample-size corrected Akaike information criteria scores (AICc and AICw) [41] provided by the *fitDiscrete* [40] and *aic.w* [42] functions, respectively. A summary of the scores obtained with all models is provided in Additional file 4: Table S2.

### Phylogenetic signal

Heritable traits (whether continuous or discrete) observed in species related by a phylogenetic history do not represent data points that are statistically independent and identically-distributed [22, 23, 43]. Hence, specific statistical methods must be applied for comparative analysis of these traits (here, nanostructures). Many indices were developed to quantify the so-called ‘phylogenetic signal’ (i.e., how much closely-related species tend to resemble each other more than randomly-drawn species) with respect to trait evolution [43, 44]. Here, we use Pagel’s λ parameter [22, 23] because of its good statistical properties (low sensitivity to tree size and to errors in topology and branch lengths, high power of associated statistical tests) over broad types of tree transformations [44]. As discussed above, with discrete traits, the λ coefficient is used in the Mk model as a scaling factor of branch lengths [22]. The *phytools* package [42] provides the function *phylosig* that tests for the presence of phylogenetic signal through randomisation of the species in the tree [22, 23, 43, 44] but cannot be used with discrete data. Hence, we follow here the proposed alternative method of Revell [45]: we optimise the Mk model likelihood under a given λ-transformation of the tree, making it an analogue of the BM model but for discrete traits, and we compare the associated estimator λ_ML_ to the null hypothesis case (λ = 0, i.e., trait evolution and phylogeny are independent). The combination of a value of λ close to 1, and significantly better likelihood than with λ = 0, indicates the presence of significant phylogenetic signal.

### Phylogenetic mapping

Using the time-calibrated tree of squamates [20] and the best fitting model of character evolution (Additional file 4: Table S2), we performed ancestral state reconstruction and substitutional history estimation of the four nano-morphological characters and the simple life habit character discussed above. The phylogenetic mapping was determined with a continuous-time reversible Markov model implemented in the function *make.simmap* of the *phytools* [42] package in R [39]. If a state could not be scored for a particular character, the corresponding species was removed from the analysis. We followed the procedure described in [42, 46, 47], and implemented in the function *make.simmap*: estimating the posterior probabilities of ancestral states by sampling the associated posterior distribution approximated by a continuous-time Markov chain. The parameters used in the Markov algorithm were: 10^4^ simulations (nsim), a burnin of 10^3^ steps and a sample frequency of 100. We used the default option of prior distribution estimation at the root by sampling the conditional scaled likelihood distribution. Then, conditioned on this prior distribution at the root and the observed states at tip branches, the mutational history of the character was simulated by sampling with the appropriate posterior distribution.

### Correlations among characters

To detect potential linear relationships (i.e.*,* correlation) between traits [19, 27, 48, 49], we performed a comparative analysis among the four nano-morphological traits and the simple classification of life habit (aquatic, terrestrial, fossorial or arboreal) using both the phylogenetic generalised least-squares regression (PGLS; [27]) and the phylogenetic generalised linear mixed model (PGLMM; [28]), two methods based on different statistical perspectives (frequentist and Bayesian inferences, respectively). Note that we do not aim to infer the best values of the model parameters but wish to identify the presence or absence of correlation. The analysis was performed on the entire 10 (5 × 4/2) pairs of characters. For each pair of characters, we pruned the global tree such that it only includes the species for which both traits have been scored.

The PGLS regression is based on a common generalised least squares (GLS) model assuming that the response variable **Y** (or any of its transformations) can be expressed by a linear relationship of explanatory variable **X**, which are independent of each other and of the error [27, 48], i.e.*,* **g(Y) = Xβ +** ∈. However, the GLS takes into account dependence of the observations (here, the species) by down-weighting the estimator with the variance-covariance matrix. Consequently, the assumption of uncorrelated errors and homoscedasticity, i.e.*,* constant variance, is relaxed. The error allows grouping within-species variation (also called measurement error), error due to unknown or incomplete phylogenetic relationships, and error due to the stochastic nature of the evolutionary process along the phylogeny [48]. As we could not estimate the error due to unknown or incomplete phylogenetic relationships and to within-species variation, we assume that the error originates only from variation of evolutionary changes along a phylogeny. This error follows a multivariate normal distribution with an expectation of **0** and variance-covariance matrix σ^2^**V**, where **V** (NxN matrix, N number of species) is known and relates to the tree structure and branch lengths. For a Brownian model, the variance is defined by **V**_ij_ = σ^2^t_ij_, where t_ij_ is the distance on the phylogeny between the root and the most recent common ancestor of taxa i and j, and σ^2^ (that can be estimated) represents the evolutionary rate [21]. One advantage of the PGLS method (function *pgls* in the *caper* package [50]) is that the matrix **V** can be adapted to multiple models of evolution by manipulating the branch lengths in the phylogenetic tree with one of Pagel’s coefficients. Note that we had to convert the discrete trait variable response into a pseudo-continuous variable in order to apply PGLS. It has been shown [26] that this approach exhibits good statistical performances and valid estimations. We used the *pgls* function with the R structure formula Var1 ~ Var2, where Var1**,** Var2 represents the tested pair of characters. Note that we fixed the parameters λ, δ, and κ to 1 as suggested by our statistical comparisons among all combinations of models (ER, SYM, ARD) and coefficients (none, λ, δ, and κ).

The PGLMM method, supported by the function *MCMCglmm* in the package of the same name [51], implements Bayesian inference with Markov Chain Monte Carlo (MCMC) approximation to fit the model. The generalised linear mixed model is an extension of the generalised linear model (with fixed **X** explanatory effects) but with the addition of random effects (denoted by the matrix **Z)** on the model. We can then write the model as **l = [X Z]ϑ +** ϵ, where **l** is the so-called latent variable (some transformation of the response variable **Y**), **X** and **Z** are the design matrices related to respectively fixed and random effects, **ϑ** (**[β a]**^**T**^) is the vector of parameters, and ϵ is the residuals’ vector. Location effects **β** (fixed), **a** (random) and residuals ϵ are assumed to be drawn from a multivariate normal distribution. While fixed effects are assumed a priori to be independently-distributed with mean **β**_0_ and variance σ_B_^2^**I**, the random effects and residuals have zero mean as well variance σ_a_^2^**A** and σ_e_^2^**I**, respectively. In phylogenetic comparative analyses, **A** is equivalent to the **V** matrix defined in PGLS. Note that σ^2^ can also represent a matrix in the case of multiple responses models (i.e., with multi-state characters), such that the global variance matrix is the Kronecker product of **σ**^**2**^ with **I** or **A**.

The main advantage of PGLMM is to model multinomial logit (=log-odds) responses (a special case of multiple response models) with more than two states, hence, it is particularly adapted to our data [28]. Multinomial logit models usually reduces the J states problem to a J-1 problem by taking one of the levels as a reference. While in PGLS the variable response corresponds exactly at the data value, multinomial PGLMM looks on log-odds ratio of the probability that a species *i* has a trait’s state *j*, i.e.*, l*_*ij*_ *= log (*α_*ij*_*/*α_*iref*_*)* where α_*ij*_ represents these probabilities and *ref* is the reference state. The *MCMCglmm* function input for the fixed effect is defined as Var1 ~ Var2 for multinomial response with 2 states (Cell shape, Ridge) and Var1 ~ trait Var2 + trait – 1 for responses with more than 2 states (Cell borders, Cell surface). The latter formulation enables to obtain specific intercepts and regression coefficients for each trait of Var1 (‘trait’ indexes the latent variables, i.e.*,* the states of the characters) with respect to Var2. The (− 1) is imposed in order to remove global intercept and to get easier interpretable models [52]. For the random effect, we define the input random ~ animal for 2 states latent variables and random ~ us(trait): animal for > 2 states latent variables. ‘Animal’ corresponds to the species identifier, while us() is an internal function that fits the variance of the random effects by assuming the complete **σ**^**2**^ (J-1) x(J-1) variance matrix of the trait, i.e.*,* covariance between levels of the trait are permitted. Note that we use the *rcov* formula for the residual covariance structure. It is defined as rcov ~ us(trait): units for > 2 states response and random ~ units for 2 states, in order to estimate the fully parametrized covariance matrix. **‘**Units’ indexes the rows of the multiple response data (here as there is only one state for each species, such that the ‘units’ index is equivalent to the ‘animal’ index).

In the context of a Bayesian framework, prior distributions should be defined. For the fixed effect, the *MCMCglmm* function assumes a multivariate normal prior [52] where the mean μ is set to **0** and the variance parameter V is set to (1.7 + π^2^/3) ***I***, where ***I*** is the (J-1) xK identity matrix (J and K are the numbers of levels for Var1 and Var2, respectively). For the random effects and the residuals, the *MCMCglmm* function assumes an inverse-Whishart prior with the parameter *V* and *ν*. Here, for the residuals, we impose V = (***I*** + ***J***) / J, where **J** is the unit matrix and *ν* = *J*-1. Additionally, we arbitrarily fix the variance throughout the MCMC run [28]. On the other hand, we can estimate the variance of the random effect during the run.

In order to increase the mixing of the MCMC, we use the parameter expansion methods [52], i.e., we add (in the prior definition of the random effects) the parameters *αμ* = 0 and *α.V* = 10^3^***I***. Other parameters of the MCMC were: 10^7^ MCMC iterations with a burnin of 10^6^ and thinning interval of 10^4^ iterations, and truncate latent variables when necessary (to prevent under/overflow). We used the *autocorr.plot* and *gewecke.plot* functions from the *coda* package [53] to evaluate Markov chain convergence [54].

## Additional files

Additional file 1: **Table S1.** Characters and their states for every species studied. Subspecies removed from the phylogenetic mapping analyses are indicated with an asterisk. Cell shape: 0 = wide, 1 = polygonal; Border elevation: 0 = levelled, 1 = elevated, 2 = high; Cell border: 0 = regular, 1 = short digits, 2 = mild digits, 3 = long digits, 4 = sawteeth; Cell surface: 0 = smooth, 1 = holes, 2 = straight channels, 3 = labyrinthine channels; Ridge: 0 = absent, 1 = present; Life habit: 0 = aquatic, 1 = terrestrial, 2 = fossorial, 3 = arboreal. The same character matrix, as well as one SEM image of the corresponding species is available in a MySQL relational database at https://snake-nanogratings.lanevol.org. (PDF 618 kb)

Additional file 2: **Movie S1.** Confocal microscopy analysis of cell border shape. Succession of confocal planes in embryonic skin of *Boaedon fuliginosus* stained with Syto9: although caudal digitations of oberhautchen cells cover and conceal the cranial edge of the next cell, the damaged portion of the sample reveals that the cranial edge (top-pointing arrow) exhibits deformations that follow the caudal digitations (bottom-pointing arrow). (AVI 376 kb)

Additional file 3: **(**Tonini_SnakesCommon.nwk) **–** The Newick tree file containing the 340 species analysed for models of character evolution, phylogenetic mapping and correlation among characters. The file was built by pruning the time-calibrated tree among 9754 species of squamates [20], keeping the 353 species for which we have obtained nanomorphological data, followed by removal of redundant subspecies. (NWK 14 kb)

Additional file 4: **Table S2.** Macroevolutionary model fitting for each character. The ‘Model’ column indicates the type of Mk model. The ‘Transformation’ and ‘Estimator’ columns indicate which of the Pagel’s tree transformation coefficient is applied and its optimised value, respectively. The last three columns represent the estimated natural log-likelihood (lnL), the sample-size corrected Akaike coefficient (AICc), and the Akaike weights (AICw), respectively. Red rows indicate the best model for each character. (PDF 486 kb)

Additional file 5: **Figure S1.** Stochastic mapping of the Cell Shape character on the full species tree. Green, ‘wide’; red, ‘polygonal’. Higher-level taxa are indicated with different colours on the corresponding branches. (PDF 1749 kb)

Additional file 6: **Figure S2.** Stochastic mapping of the Cell Border character on the full species tree. Red, ‘regular’; light green, ‘short digits’; mild green, ‘mild digits’; dark green, ‘long digits’; blue, ‘sawteeth’. Higher-level taxa are indicated with different colours on the corresponding branches. (PDF 2299 kb)

Additional file 7: **Figure S3.** Comparison between two types of digitations. Digits with **(a)** sharp tips in *Boaedon fuliginosus* (a representative species of Caenophidia) and **(b)** round tips in *Morelia spilota spilota* (a representative of Pythonidae). Scale bars: 5 μm. (PDF 8187 kb)

Additional file 8: **Figure S4.** Stochastic mapping of the Cell Surface character on the full species tree. Red, ‘smooth’; green, ‘holes’; blue, ‘straight channels’; yellow, ‘labyrinthine channels’. Asterisks indicate species categorised as ‘smooth’ although they possess very small depressions that do not pass the algorithm’s requirements to be classified as ‘holes’. Higher-level taxa are indicated with different colours on the corresponding branches. (PDF 2359 kb)

Additional file 9: **Figure S5.** Parameter distributions across all species for categorisation of cell surface structures. **(a)** Distribution of bounding rectangle length/width ratio (rounded at the nearest integer) for the differentiation of holes and channels. Vertical blue line: arbitrary threshold. **(b)** Channel angle sorted standard deviations. The chosen threshold to differentiate straight from labyrinthine channels is set at 25° (red line), i.e., within the largest interval of unobserved SD values. (PDF 158 kb)

Additional file 10: **Figure S6.** Cell surface state distribution. Distribution across species of the ratios between the surfaces of ‘holes’ and ‘straight channels’ observed within a species. The threshold separating ‘straight channels’ and ‘holes’ corresponds to a ratio of 1.0. (PDF 21 kb)

Additional file 11: **Figure S7.** Assignment of the cell surface state. **(a)** ‘holes’; **(b)** ambiguity between ‘holes’ and ‘labyrinthine channels’; **(c)** ‘labyrinthine channels’. Scale bars: 2 μm. (PNG 721 kb)

Additional file 12: **Figure S8.** Stochastic mapping of the Ridge character on the full species tree. Green, ‘absence’; red, ‘presence’. Higher-level taxa are indicated with different colours on the corresponding branches. (PDF 329 kb)

Additional file 13: **Figure S9.** Stochastic mapping of the Life Habit character on the full species tree. Red, ‘aquatic’; dark green, ‘terrestrial’; blue, ‘fossorial’; yellow, ‘arboreal’; orange, ‘aquatic + terrestrial’; turquoise, ‘terrestrial + fossorial’; light green, ‘terrestrial + arboreal’. Higher-level taxa are indicated with different colours on the corresponding branches. (PDF 328 kb)

Additional file 14: **Figure S10.** Highly-organised nanostructures. **(a)** Digitations in *Xenopeltis unicolor* and **(b)** cell surface ‘holes’ in *Boiga multimaculata.* Scale bars: 2 μm (a) and 5 μm (b). (PDF 4238 kb)

Additional file 15: **Figure S11.** Identification of cell surface structures using image analysis. **(a)** Original SEM image; **(b)** Contrast Limited Adaptive Histogram Equalisation; **(c)** local k-means pixel clustering into three categories based on their intensity (black, grey and white); **(d)** identification of the darkest pixels that form contours; **(e)** grouping the contours into four classes: holes (blue), straight channels (red), labyrinthine channels (green) and unclassified (yellow); **(f)** final classification after manual correction. (PDF 2785 kb)

Additional file 16: Summary statistics for the phylogenetic generalised least squares regression (PGLS). (TXT 8 kb)

Additional file 17: Summary statistics for the phylogenetic generalised linear mixed models (PGLMM). (TXT 22 kb)

## Abbreviations

AICc: Sample-size corrected Akaike information criterion
AICw: Normalised Akaike weights
ARD: Model with all rates different
ASR: Area, solidity and contour-surface-to-bounding-convex-polygon-surface ratio
ER: Equal rate model
MCMC: Markov Chain Monte Carlo approximation
Mk models: Markov-chain models
ML: Maximum likelihood
PGLMM: Phylogenetic generalised linear mixed model
PGLS: Phylogenetic generalised least squares regression approach
SEM: Scanning electron microscopy
SYM: Symmetrical rate model
