# Supplementary material for: Phylogenetic mapping of scale nanostructure diversity in snakes
Source: BMC Evol Biol. 2019 Apr 16;19:91. doi: 10.1186/s12862-019-1411-6 (PMC6469093; doi:10.1186/s12862-019-1411-6)
Supplement: Supplementary file 1 — Table S1. Characters and their states for every species studied. Subspecies removed from the phylogenetic mapping analyses are indicated with an asterisk. Cell shape: 0 = wide, 1 = polygonal; Border elevation: 0 = levelled, 1 = elevated, 2 = high; Cell border: 0 = regular, 1 = short digits, 2 = mild digits, 3 = long digits, 4 = sawteeth; Cell surface: 0 = smooth, 1 = holes, 2 = straight channels, 3 = labyrinthine channels; Ridge: 0 = absent, 1 = present; Life habit: 0 = aquatic, 1 = terrestrial, 2 = fossorial, 3 = arboreal. The same character matrix, as well as one SEM image of the corresponding species is available in a MySQL relational database at https://snake-nanogratings.lanevol.org. (PDF 618 kb) [file 12862_2019_1411_MOESM1_ESM.pdf]

| Species | Cell shape | Cell border | Cell surface | Digit fibre | Life habit |
|---|---|---|---|---|---|
| Trilepida macrolepis | 0 | 1 | 0 | 0 | 2 |
| Liotyphlops ternetzii | 0 | 1 | 0 | 0 | 2 |
| Acutotyphlops subocularis | 0 | 0 | 0 | 0 | 2 |
| Argyrophis diardii | 0 | 0 | 0 | 0 | 2 |
| Indotyphlops braminus | 0 | 0 | 0 | 0 | 2 |
| Gerrhopilus mirus | N/A | 0 | 0 | 0 | 2 |
| Grypotyphlops acutus | N/A | 0 | 0 | 0 | 2 |
| Xerotyphlops vermicularis | 0 | 1 | 0 | 0 | 2 |
| Anilius scytale | 0 | 2 | 0 | 0 | 2 |
| Anomochilus leonardi | 0 | 2 | 0 | 0 | 2 |
| Cylindrophis maculatus | 0 | 2 | 0 | 0 | 1 + 2 |
| Cylindrophis ruffus | 0 | 1 | 0 | 0 | 1 + 2 |
| Brachyophidium rhodogaster | 0 | 2 | 0 | 0 | 2 |
| Melanophidium bilineatum | 1 | 0 | 1 | 0 | 2 |
| Melanophidium punctatum | 1 | 0 | 1 | 0 | 2 |
| Melanophidium wynaudense | 1 | 0 | 1 | 0 | 2 |
| Plectrurus perroteti | 0 | 0 | 1 | 0 | 2 |
| Platyplectrurus trilineatus | 0 | 3 | 0 | 0 | 2 |
| Pseudotyphlops philippinus | 0 | 1 | 1 | 0 | 2 |
| Rhinophis blythii | 0 | 1 | 2 | 0 | 2 |
| Rhinophis drummondhayi | 0 | 1 | 1 | 0 | 2 |
| Rhinophis homolepis | 0 | 1 | 1 | 0 | 2 |
| Rhinophis oxyrhynchus | 0 | 1 | 1 | 0 | 2 |
| Rhinophis philippinus | 0 | 1 | 1 | 0 | 2 |
| Rhinophis sanguineus | 0 | 1 | 1 | 0 | 2 |
| Rhinophis travancoricus | 0 | 1 | 1 | 0 | 2 |
| Teretrurus sanguineus | 0 | 2 | 1 | 0 | 2 |
| Uropeltis ceylanicus | 0 | 2 | N/A | 0 | 2 |
| Uropeltis ellioti | 0 | 1 | 1 | 0 | 2 |
| Uropeltis macrolepis | 0 | 2 | 1 | 0 | 2 |
| Uropeltis melanogaster | 0 | 1 | 1 | 0 | 2 |
| Uropeltis phillipsi | 0 | 1 | 1 | 0 | 2 |
| Uropeltis phipsonii | 0 | 2 | 1 | 0 | 2 |
| Xenopeltis unicolor | 0 | 2 | 0 | 0 | 2 |
| Antaresia maculosa | 0 | 3 | 0 | 0 | 1 |
| Aspidites melanocephalus | 0 | 3 | 0 | 0 | 1 |
| Aspidites ramsayi | 0 | 1 | 0 | 0 | 1 |
| Bothrochilus boa | 0 | 2 | 0 | 0 | 1 |
| Bothrochilus albertisii | 0 | 2 | 0 | 0 | 1 |
| Liasis fuscus | 0 | 3 | N/A | 0 | 1 + 0 |
| Liasis mackloti dunni | 0 | 3 | 0 | 0 | 1 + 0 |
| Liasis mackloti mackloti* | 0 | 3 | 1 | 0 | 1 + 0 |
| Liasis papuana | 0 | 2 | 0 | 0 | 1 |

| | | | | | |
|---|---|---|---|---|---|
| Morelia spilota harrisoni* | 0 | 3 | 1 | 0 | 3 |
| Morelia spilota spilota | 0 | 3 | 2 | 0 | 3 |
| Morelia viridis | 0 | 2 | 1 | 0 | 3 |
| Python breitensteini | 0 | 1 | 1 | 0 | 1 |
| Python brongersmai | 0 | 1 | 0 | 0 | 1 |
| Python bivittatus | 0 | 2 | 2 | 0 | 1 |
| Python regius | 0 | 2 | 1 | 0 | 1 + 2 |
| Python sebae | 0 | 2 | 2 | 0 | 1 |
| Simalia amethistina | 0 | 3 | N/A | 0 | 3 |
| Simalia boeleni | 0 | 2 | 0 | 0 | 3 |
| Boa constrictor imperator* | N/A | 0 | 1 | 0 | 1 + 3 |
| Boa constrictor sabogae | 1 | 0 | 2 | 0 | 1 + 3 |
| Corallus annulatus | N/A | 0 | 1 | 0 | 3 |
| Corallus caninus | N/A | 0 | 2 | 0 | 3 |
| Corallus cookii | N/A | 0 | 1 | 0 | 3 |
| Corallus hortulanus | N/A | 0 | 1 | 0 | 3 |
| Corallus ruschenbergerii | N/A | 0 | 2 | 0 | 3 |
| Chilabothrus angulifer | 1 | 4 | 2 | 0 | 3 |
| Chilabothrus fordii | 1 | 4 | 2 | 0 | 3 |
| Chilabothrus inornatus | 0 | 4 | 2 | 0 | 3 |
| Chilabothrus striatus | 1 | 4 | 1 | 0 | 3 |
| Chilabothrus strigilatus | 1 | 4 | 1 | 0 | 3 |
| Chilabothrus subflavus | 1 | 4 | 1 | 0 | 3 |
| Epicrates alvarezi | 1 | 4 | 1 | 0 | 3 |
| Epicrates cenchria | 1 | 4 | 2 | 0 | 1 + 3 |
| Epicrates crassus | 1 | 4 | 2 | 0 | 1 + 3 |
| Epicrates maurus | 0 | 4 | 1 | 0 | 1 + 3 |
| Eunectes murinus | 0 | 4 | 2 | 0 | 1 + 0 |
| Eunectes notaeus | 0 | 4 | 2 | 0 | 1 + 0 |
| Eryx conicus | 1 | 0 | 1 | 0 | 2 |
| Eryx colubrinus loveridgei | N/A | 0 | N/A | 0 | 2 |
| Eryx jaculus turcicus | N/A | N/A | 1 | 0 | 2 |
| Candoia bibroni australis | N/A | 0 | N/A | 0 | 1 + 3 |
| Candoia carinata | N/A | 0 | 1 | 0 | 1 + 3 |
| Lichanura trivirgata saslowi | 0 | 1 | 0 | 0 | 1 + 2 |
| Charina bottae | 0 | 4 | 0 | 0 | 1 + 2 |
| Acrantophis dumerili | N/A | 4 | 2 | 0 | 1 + 3 |
| Acrantophis madagascariensis | N/A | N/A | 2 | 0 | 1 + 3 |
| Sanzinia madagascariensis | N/A | 4 | 1 | 0 | 3 |
| Calabaria reinhardtii | 0 | 1 | 2 | 0 | 1 + 2 |
| Ungaliophis panamensis | 1 | 4 | 1 | 0 | 2 |
| Casarea dussumieri | N/A | 0 | 1 | 0 | 1 + 2 |
| Acrochordus granulatus | N/A | N/A | N/A | 0 | 0 |
| Acrochordus javanicus | N/A | N/A | N/A | 0 | 0 |

| | | | | | |
|---|---|---|---|---|---|
| Xylophis perroteti | 0 | 3 | N/A | 0 | 1 |
| Aplopeltura boa | 1 | 0 | 1 | 0 | 3 |
| Pareas carinatus | N/A | N/A | 1 | 0 | 3 |
| Pareas monticola | N/A | 1 | 1 | 0 | 3 |
| Agkistrodon contortrix | N/A | 1 | 1 | 0 | 1 |
| Agkistrodon piscivorus | N/A | N/A | 1 | 0 | 1 + 0 |
| Atropoides nummifer mexicanus | 1 | 0 | 2 | 0 | 1 |
| Atropoides olmec | 1 | 0 | 3 | 0 | 1 |
| Bothriechis aurifer | N/A | 0 | 1 | 0 | 3 |
| Bothriechis bicolor | N/A | 0 | 1 | 0 | 3 |
| Bothriechis marchi | N/A | 0 | 1 | 0 | 3 |
| Bothriechis nigroviridis | N/A | 0 | 1 | 0 | 3 |
| Bothriechis schlegelii | N/A | 0 | 1 | 0 | 3 |
| Bothrops alternatus | N/A | 0 | 1 | 0 | 1 |
| Bothrops asper | 1 | 0 | 1 | 0 | 3 |
| Bothrops atrox | 1 | 0 | 1 | 0 | 1 |
| Bothrops bilineata | N/A | 0 | 1 | 0 | 3 |
| Bothrops jararacussu | 1 | 0 | 1 | 0 | 1 |
| Bothrops pictus | 1 | 0 | 1 | 0 | 1 |
| Calloselasma rhodostoma | N/A | N/A | 1 | 0 | 1 |
| Crotalus adamanteus | 1 | 0 | 2 | 0 | 1 |
| Crotalus atrox | 1 | 0 | 2 | 0 | 1 |
| Crotalus cerastes | 0 | 2 | 2 | 1 | 1 |
| Crotalus durissus vergrandis | 1 | 0 | 3 | 0 | 1 |
| Crotalus durissus pifanorum* | N/A | 0 | 1 | 0 | 1 |
| Crotalus simus | 1 | 0 | 1 | 0 | 1 |
| Crotalus viridis | 1 | 1 | 2 | 0 | 1 |
| Gloydius brevicaudus | N/A | 1 | 3 | 0 | 1 |
| Gloydius himalayanus | N/A | N/A | 2 | 0 | 1 |
| Hypnale hypnale | N/A | 0 | 1 | 0 | 1 + 3 |
| Lachesis melanocephala | N/A | 0 | 1 | 0 | 1 |
| Lachesis stenophrys | N/A | N/A | 1 | 0 | 1 |
| Mixcoatlus melanurus | N/A | 1 | 2 | 0 | 1 |
| Ovophis monticola | N/A | 0 | 1 | 0 | 1 |
| Porthidium lansbergii rozei | 1 | 0 | 1 | 0 | 1 |
| Porthidium nasutum | 1 | 0 | 1 | 0 | 1 |
| Porthidium ophryomegas | 1 | 0 | 1 | 0 | 1 |
| Porthidium porrasi | 1 | 0 | 1 | 0 | 1 |
| Porthidium volcanicum | 1 | 0 | 1 | 0 | 1 |
| Protobothrops jerdonii | N/A | 0 | 1 | 0 | 1 + 3 |
| Protobothrops mangshanensis | N/A | 1 | 1 | 0 | 3 |
| Protobothrops mucrosquamatus | N/A | 0 | 2 | 0 | 1 + 3 |

| | | | | | |
|---|---|---|---|---|---|
| Sistrurus miliarius | N/A | 0 | 1 | 0 | 1 |
| Trimeresurus albolabris | 1 | 0 | 1 | 0 | 1 + 3 |
| Trimeresurus erythrurus | N/A | N/A | 2 | 0 | 1 + 3 |
| Trimeresurus gramineus | N/A | 0 | 2 | 0 | 3 |
| Trimeresurus labialis | N/A | 0 | 1 | 0 | 1 |
| Trimeresurus macrolepis | N/A | 0 | 1 | 0 | 1 + 3 |
| Trimeresurus macrops | 1 | 0 | 1 | 0 | 3 |
| Trimeresurus malabaricus | N/A | 0 | 2 | 0 | 3 |
| Trimeresurus popeiorum | 1 | 0 | 2 | 0 | 1 + 3 |
| Trimeresurus purpureomaculatus | N/A | N/A | 1 | 0 | 3 |
| Trimeresurus strigatus | N/A | N/A | N/A | 0 | 1 |
| Trimeresurus trigonocephalus | N/A | 0 | 1 | 0 | 3 |
| Trimeresurus kanburiensis | N/A | N/A | 1 | 0 | 3 |
| Tropidolaemus wagleri | N/A | N/A | 0 | 0 | 3 |
| Atheris desaixi | 1 | 0 | 1 | 0 | 3 |
| Atheris hispida | 1 | 0 | 1 | 0 | 3 |
| Atheris nitschei | 1 | 0 | 1 | 0 | 3 |
| Atheris squamigera | 1 | 0 | 1 | 0 | 3 |
| Bitis arietans | N/A | 0 | 0 | 0 | 1 |
| Bitis caudalis | 1 | 0 | 1 | 0 | 1 |
| Bitis gabonica rhinoceros | 1 | 0 | 3 | 0 | 1 |
| Bitis nasicornis | 1 | 0 | 3 | 0 | 1 |
| Causus bilineatus | N/A | N/A | N/A | 0 | 1 |
| Causus maculatus | N/A | 3 | N/A | 0 | 1 |
| Causus resimus | N/A | N/A | N/A | 0 | 1 |
| Cerastes cerastes | 1 | 0 | 1 | 0 | 1 |
| Daboia palaestinae | N/A | 1 | 2 | 0 | 1 |
| Daboia russelii | 1 | 0 | 1 | 0 | 1 |
| Echis carinatus carinatus* | N/A | 0 | 1 | 0 | 1 |
| Echis carinatus sochureki | 1 | 0 | 3 | 0 | 1 |
| Echis coloratus | 1 | 0 | 1 | 0 | 1 |
| Echis ocellatus | 1 | 0 | 1 | 0 | 1 |
| Macrovipera lebetina | N/A | 0 | 3 | 0 | 1 |
| Montivipera xanthina | N/A | N/A | 2 | 0 | 1 |
| Proatheris superciliaris | 1 | 0 | 1 | 0 | 1 |
| Pseudocerastes persicus | 1 | 0 | 1 | 0 | 1 |
| Vipera ammodytes | 0 | 1 | 2 | 0 | 1 |
| Vipera aspis | N/A | 0 | 2 | 0 | 1 |
| Vipera berus | 0 | 0 | 2 | 0 | 1 |
| Dieurostus dussumieri | 0 | 2 | 0 | 0 | 0 |
| Hypsiscopus plumbea | 0 | 3 | 0 | 0 | 0 |
| Ferania sieboldii | 0 | 1 | 2 | 0 | 0 |
| Gerarda prevostiana | 1? | 0 | 1 | 0 | 0 |

| | | | | | |
|---|---|---|---|---|---|
| Acanthophis antarcticus | N/A | 1 | 1 | 0 | 1 |
| Acanthophis hawkei | 0 | 2 | 2 | 0 | 1 |
| Acanthophis laevis | 0 | 1 | 2 | 0 | 1 |
| Aipysurus fuscus | 1 | 0 | 0 | 0 | 0 |
| Aspidelaps lubricus | 0 | 2 | 2 | 0 | 1 |
| Aspidelaps scutatus | 0 | 1 | 2 | 0 | 2 |
| Aspidomorphus muelleri | 0 | 3 | 2 | 0 | 1 |
| Aspidomorphus lineaticollis | 0 | 3 | 2 | 0 | 1 |
| Bungarus caeruleus | 0 | 2 | 0 | 0 | 1 |
| Bungarus fasciatus | 0 | 2 | 0 | 0 | 1 |
| Bungarus flaviceps | 0 | 3 | 0 | 0 | 1 |
| Calliophis bibroni | 0 | 2 | 2 | 0 | 1 |
| Calliophis bivirgata | 0 | 3 | 2 | 0 | 1 |
| Calliophis nigrescens | 0 | 3 | 2 | 0 | 1 |
| Dendroaspis angusticeps | N/A | N/A | N/A | 0 | 3 |
| Dendroaspis jamesoni kaimosae | N/A | N/A | N/A | 0 | 3 |
| Dendroaspis polylepis | N/A | N/A | N/A | 0 | 1 |
| Elapsoidea semiannulata | 0 | 2 | 0 | 0 | 2 |
| Hemachatus haemachatus | 0 | 2 | 3 | 1 | 1 |
| Hydrophis cyanocinctus | 1 | 0 | 0 | 0 | 0 |
| Hydrophis lapemoides | 1 | 0 | 0 | 0 | 0 |
| Laticauda colubrina | 1 | 1 | 0 | 0 | 0 |
| Micropechis ikaheca | 0 | 2 | 0 | 0 | 1 + 2 |
| Micrurus corallinus | 0 | 3 | 2 | 0 | 2 |
| Micrurus mipartitus | 0 | 2 | 0 | 0 | 1 |
| Micrurus nigrocinctus | 0 | 2 | 0 | 0 | 1 |
| Naja annulata | 0 | 2 | 2 | 0 | 0 |
| Naja annulifera | 0 | 2 | 1 | 1 | 1 |
| Naja atra | 0 | 1 | 2 | 0 | 1 |
| Naja haje legionis | 0 | 2 | 1 | 0 | 1 |
| Naja kaouthia | 0 | 3 | 2 | 0 | 1 |
| Naja melanoleuca | 0 | 2 | 0 | 0 | 1 |
| Naja naja | 0 | 3 | 2 | 0 | 1 |
| Naja nigricollis | 0 | 2 | 1 | 0 | 1 |
| Naja nivea | 0 | 2 | 0 | 0 | 1 |
| Naja oxiana | 0 | 3 | 0 | 0 | 1 |
| Naja pallida | 0 | 2 | 2 | 0 | 1 |
| Naja samarensis | 0 | 2 | 2 | 0 | 1 |
| Naja siamensis | 0 | 2 | 2 | 0 | 1 |
| Notechis scutatus | 0 | 2 | N/A | 1 | 1 |
| Ophiophagus hannah | 0 | 3 | 2 | 0 | 1 |
| Oxyuranus microlepidotus | 0 | 3 | 2 | 1 | 1 |
| Oxyuranus scutellatus canni | 0 | 2 | 2 | 0 | 1 |
| Pseudohaje goldii | 0 | 2 | 0 | 0 | 3 |

| | | | | | |
|---|---|---|---|---|---|
| Toxicocalamus loriae | 0 | 3 | 2 | 0 | 2 |
| Boaedon fuliginosus | 0 | 3 | 2 | 0 | 1 |
| Gonionotophis poensis | 0 | 2 | 0 | 0 | 1 |
| Lycophidion albomaculatum | 0 | 2 | 0 | 0 | 1 |
| Oxyrhabdium modestum | 0 | 1 | 1 | 0 | 1 |
| Atractaspis bibronii | 0 | 3 | 0 | 0 | 2 |
| Malpolon insignitus | 0 | 3 | 2 | 1 | 1 |
| Psammodynastes pictus | N/A | N/A | N/A | 0 | 1 |
| Psammodynastes pulverulentus | 0 | 2 | 1 | 1 | 1 |
| Psammophis schokari | 0 | 3 | N/A | 1 | 1 |
| Psammophis sibilans | 0 | 3 | 2 | 1 | 1 |
| Rhamphiophis rostratus | 0 | 3 | 2 | 1 | 1 |
| Liopholidophis sexlineatus | 0 | 2 | 2 | 0 | 0 |
| Ahaetulla dispar | N/A | 0 | 1 | 1 | 3 |
| Ahaetulla nasuta | N/A | 3 | 1 | 1 | 3 |
| Ahaetulla perroteti | N/A | 3 | 1 | 1 | 3 |
| Ahaetulla prasina | N/A | N/A | N/A | 0 | 3 |
| Ahaetulla pulverulenta | N/A | 3 | 1 | 1 | 3 |
| Bogertophis subocularis | 0 | 3 | 2 | 0 | 1 |
| Boiga ceylonensis | N/A | N/A | 1 | 0 | 3 |
| Boiga cyanea | 0 | 1 | 2 | 0 | 1 + 3 |
| Boiga cynodon | 0 | 1 | 1 | 0 | 1 + 3 |
| Boiga dendrophila gemmicincta | 0 | 2 | 2 | 0 | 3 |
| Boiga forsteni | 0 | 1 | 2 | 0 | 3 |
| Boiga gokool | 1 | 1 | 1 | 0 | 3 |
| Boiga irregularis | 0 | 2 | 2 | 0 | 3 |
| Boiga multomaculata | 0 | 1 | 1 | 0 | 3 |
| Boiga nigriceps | N/A | 1 | 1 | 0 | 3 |
| Boiga siamensis | N/A | N/A | 1 | 0 | 3 |
| Boiga ochracea | N/A | 1 | 1 | 0 | 3 |
| Boiga tanahjampeana | 0 | 1 | 2 | 0 | 3 |
| Boiga trigonata | 1 | 1 | 1 | 0 | 3 |
| Boiga wallachi | 0 | 1 | 2 | 0 | 1 + 3 |
| Chironius multiventris | 0 | 3 | 2 | 1 | 1 + 3 |
| Chrysopelea ornata | 0 | 3 | 0 | 1 | 3 |
| Chrysopelea paradisi | 0 | 3 | 2 | 1 | 3 |
| Chrysopelea pelias | 0 | 3 | 2 | 1 | 3 |
| Chrysopelea rhodopleuron | 0 | 3 | 2 | 1 | 3 |
| Coelognathus helena helena | 0 | 3 | 2 | 0 | 1 |
| Coelognathus helena monticollaris* | 0 | 3 | 2 | 0 | 1 |
| Coelognathus radiatus | 0 | 2 | 2 | 0 | 1 |
| Crotaphopeltis hotamboeia | N/A | N/A | 2 | 0 | 1 |
| Dasypeltis palmarum | N/A | 0 | 1 | 0 | 1 |

| | | | | | |
|---|---|---|---|---|---|
| Dasypeltis scabra | N/A | 0 | 1 | 0 | 1 |
| Dendrelaphis ashoki | 0 | 3 | 1 | 1 | 3 |
| Dendrelaphis pictus | 0 | 3 | 1 | 1 | 1 + 3 |
| Dendrelaphis punctulatus | 0 | 2 | 2 | 1 | 3 |
| Dendrelaphis tristis | 0 | 2 | 2 | 1 | 3 |
| Dipsadoboa viridis | 0 | 1 | 1 | 0 | 1 + 3 |
| Dispholidus typus | 0 | 2 | 3 | 1 | 3 |
| Drymarchon corais corais | 0 | 2 | 2 | 0 | 1 |
| Drymarchon couperi | 0 | 3 | 0 | 0 | 1 |
| Drymarchon melanurus | 0 | 2 | 0 | 0 | 1 |
| Dryocalamus davisonii | 0 | 3 | 0 | 1 | 1 |
| Elachistodon westermanni | 0 | 1 | 1 | 0 | 1 |
| Euprepiophis mandarinus | 0 | 3 | 0 | 0 | 1 |
| Gonyosoma oxycephalum | 0 | 3 | 1 | 1 | 3 |
| Hapsidophrys smaragdinus | N/A | N/A | N/A | 0 | 3 |
| Lampropeltis getula californiae | 0 | 2 | 2 | 0 | 1 |
| Lampropeltis triangulum gaigeae* | 0 | 3 | 2 | 0 | 1 |
| Lampropeltis triangulum hondurensis* | 0 | 2 | 2 | 0 | 1 |
| Lampropeltis triangulum sinaloae | 0 | 2 | 0 | 0 | 1 |
| Lampropeltis triangulum stuarti* | 0 | 2 | 0 | 0 | 1 |
| Leptophis ahaetulla | 0 | 3 | 3 | 1 | 3 |
| Leptophis riveti | 0 | 2 | 2 | 1 | 3 |
| Lycodon rufozonatus | 0 | 3 | 0 | 0 | 1 |
| Lycodon subcinctus | 0 | 1 | 2 | 0 | 1 |
| Lytorhynchus diadema | 0 | 1 | 2 | 1 | 1 |
| Oligodon maculatus | 0 | 2 | 2 | 0 | 1 |
| Oligodon taeniolatus fasciatus | 0 | 3 | N/A | 0 | 1 |
| Oreocryptophis porphyraceus vaillanti | 0 | 2 | 2 | 0 | 1 |
| Orthriophis cantoris | 0 | 2 | 2 | 0 | 1 |
| Orthriophis moellendorffi | 0 | 2 | 2 | 0 | 1 |
| Orthriophis taeniurus friesi | 0 | 2 | 2 | 0 | 1 |
| Orthriophis taeniurus callicyanous* | 0 | 2 | 2 | 0 | 1 |
| Oxybelis aeneus | 0 | 1 | 2 | 1 | 3 |
| Oxybelis fulgidus | 0 | 2 | N/A | 1 | 3 |
| Pantherophis guttatus | 0 | 2 | 2 | 0 | 1 |
| Pantherophis obsoletus quadrivittata* | 0 | 1 | 2 | 0 | 1 |
| Pantherophis obsoletus | 0 | 2 | 2 | 0 | 1 |

| | | | | | |
|---|---|---|---|---|---|
| lindheimeri | | | | | |
| Philothamnus angolensis | 0 | 2 | 2 | 1 | 3 |
| Philothamnus hoplogaster | 0 | 3 | 2 | 1 | 1 |
| Phryonax poecilonotus | 0 | 2 | 2 | 0 | 1 |
| Platyceps ventromaculatus | 0 | 1 | 2 | 0 | 1 |
| Pseudelaphe flavirufa | 0 | 3 | 2 | 0 | 1 |
| Ptyas korros | 0 | 2 | 0 | 0 | 1 + 3 |
| Rhinobothryum bovallii | 0 | 2 | 2 | 0 | 1 |
| Senticolis triaspis | 0 | 2 | 2 | 0 | 1 |
| Spalerosophis diadema | 0 | 1 | 1 | 0 | 1 |
| Spilotes pullatus | 0 | 1 | 1 | 1 | 1 |
| Stegonotus modestus | 0 | 3 | N/A | 0 | 1 + 2 |
| Telescopus fallax | N/A | 1 | 2 | 0 | 1 |
| Telescopus rhinopoma | N/A | 1 | 2 | 0 | 1 |
| Telescopus semiannulatus | N/A | 1 | 2 | 0 | 1 |
| Thelotornis capensis | 0 | 3 | 1 | 1 | 3 |
| Thelotornis kirtlandii | 0 | 3 | 1 | 1 | 3 |
| Toxicodryas blandingii | N/A | N/A | N/A | 0 | 3 |
| Toxicodryas pulverulenta | N/A | N/A | N/A | 0 | 3 |
| Trimorphodon biscutatus | 0 | 1 | 2 | 0 | 1 |
| Zamenis longissimus | 0 | 3 | 0 | 0 | 1 |
| Zamenis situla | 0 | 2 | 2 | 0 | 1 |
| Conophis lineatus | 0 | 3 | 2 | 1 | 1 |
| Dipsas albifrons | N/A | 1 | 1 | 0 | 3 |
| Dipsas incerta | 0 | 1 | 1 | 0 | 3 |
| Erythrolamprus poecilogyrus sublineatus | 0 | 3 | 2 | 0 | 1 + 0 |
| Helicops carinicaudus | N/A | 2 | 2 | 0 | 0 |
| Helicops trivittatus | 0 | 2 | 1 | 0 | 0 |
| Heterodon nasicus | 0 | 2 | 2 | 1 | 1 |
| Imantodes cenchoa | 0 | 3 | 2 | 1 | 3 |
| Leptodeira annulata | 0 | 2 | 1 | 0 | 3 |
| Leptodeira septentrionalis | 0 | 2 | 2 | 0 | 1 |
| Oxyrhopus melanogenys | 0 | 3 | 0 | 0 | 1 |
| Oxyrhopus rhombifer bachmani* | 0 | 2 | 2 | 0 | 1 |
| Oxyrhopus rhombifer inaequifasciatus* | 0 | 3 | 2 | 0 | 1 |
| Oxyrhopus rhombifer rhombifer | 0 | 3 | 2 | 0 | 1 |
| Philodryas baroni | 0 | 1 | 2 | 1 | 3 |
| Philodryas chamissonis | 0 | 2 | 1 | 0 | 1 |
| Philodryas patagoniensis | 0 | 2 | 2 | 1 | 1 |
| Xenodon dorbignyi | N/A | 3 | 2 | 0 | 1 |
| Xenodon semicinctus | 0 | 2 | 2 | 0 | 1 |

| | | | | | |
|---|---|---|---|---|---|
| Amphiesma stolatum | 0 | 1 | 2 | 0 | 1 |
| Hebius beddomei | N/A | 1 | 1 | 0 | 1 |
| Hebius monticola | N/A | N/A | 1 | 0 | 1 |
| Hebius venningi | N/A | 1 | 1 | 0 | 1 |
| Macropisthodon plumbicolor | N/A | 0 | 1 | 0 | 1 |
| Natriciteres fuliginoides | 0 | 2 | 2 | 1 | 1 + 0 |
| Natrix maura | 0 | 1 | 0 | 1 | 1 + 0 |
| Natrix natrix | 0 | 1 | 1 | 1 | 1 + 0 |
| Nerodia clarkii | 0 | 2 | 2 | 0 | 1 + 0 |
| Regina grahamii | N/A | 3 | N/A | 1 | 0 |
| Rhabdophis himalayanus | N/A | 0 | 1 | 0 | 1 |
| Sibynophis collaris | 0 | 2 | 2 | 1 | 1 |
| Trachischium guentheri | 0 | 2 | 1 | 0 | N/A |
| Trachischium tenuiceps | 0 | 1 | 1 | 0 | N/A |
| Xenochrophis piscator | 0 | 1 | 2 | 0 | 1 |
| Grayia smithii | 0 | 3 | 0 | 0 | 0 |
| Pseudoxenodon macrops | 0 | 1 | 1 | 0 | 1 |
