# Supplementary material for: Phylogenetic mapping of scale nanostructure diversity in snakes
Source: BMC Evol Biol. 2019 Apr 16;19:91. doi: 10.1186/s12862-019-1411-6 (PMC6469093; doi:10.1186/s12862-019-1411-6)
Supplement: Supplementary file 4 — Table S2. Macroevolutionary model fitting for each character. The ‘Model’ column indicates the type of Mk model. The ‘Transformation’ and ‘Estimator’ columns indicate which of the Pagel’s tree transformation coefficient is applied and its optimised value, respectively. The last three columns represent the estimated natural log-likelihood (lnL), the sample-size corrected Akaike coefficient (AICc), and the Akaike weights (AICw), respectively. Red rows indicate the best model for each character. (PDF 486 kb) [file 12862_2019_1411_MOESM4_ESM.pdf]

Character	Model	Transformation	Estimator	lnL	AICc	AICw
Cell Shape						
	ER	-	-	-65.344645	132.7054	0.08949449
	ER	λ	0.968842	-64.644242	133.3371	0.06525831
	ER	δ	1.185435	-65.322801	134.6942	0.03310865
	ER	κ	0.671273	-64.837786	133.7242	0.05377504
	SYM	-	-	-65.344645	132.7054	0.08949449
	SYM	λ	0.968842	-64.644242	133.3371	0.06525831
	SYM	δ	1.185427	-65.322801	134.6942	0.03310865
	SYM	κ	0.671273	-64.837786	133.7242	0.05377504
	ARD	-	-	-63.549283	131.1471	0.19506093
	ARD	λ	0.973859	-62.962196	132.0220	0.12595324
	ARD	δ	0.006738	-63.088507	132.2746	0.11100770
	ARD	κ	0.770802	-63.358930	132.8154	0.08470515
Cell Border						
	ER	-	-	-365.483456	732.9799	0.00000000
	ER	λ	0.963293	-362.976099	729.9914	0.00000000
	ER	δ	1.421928	-365.264820	734.5689	0.00000000
	ER	κ	0.710275	-363.857102	731.7534	0.00000000
	SYM	-	-	-299.595113	619.9285	0.21590736
	SYM	λ	0.966427	-297.545857	617.9806	0.57179959
	SYM	δ	0.664978	-299.332818	621.5545	0.09575831
	SYM	κ	0.774488	-299.193246	621.2754	0.11010115
	ARD	-	-	-292.885975	628.6886	0.00270413
	ARD	λ	0.987732	-292.191745	629.6030	0.00171187
	ARD	δ	0.715910	-292.578702	630.3769	0.00116257
	ARD	κ	1.000000	-292.885975	630.9915	0.00085501
Cell Surface						
	ER	-	-	-355.132013	712.2770	0.00000000
	ER	λ	0.899877	-348.492981	701.0250	0.00000000
	ER	δ	2.410202	-353.290692	710.6205	0.00000000
	ER	κ	0.081364	-347.159830	698.3587	0.00000000
	SYM	-	-	-319.943536	652.1643	0.00236106
	SYM	λ	0.889429	-313.398189	641.1672	0.57688401
	SYM	δ	1.940929	-319.269430	652.9097	0.00162645
	SYM	κ	0.519162	-315.364496	645.0999	0.08074805
	ARD	-	-	-312.028937	649.1084	0.01088157
	ARD	λ	0.925272	-307.619994	642.4697	0.30078679
	ARD	δ	0.788941	-311.955206	651.1401	0.00394003
	ARD	κ	0.522019	-310.200862	647.6315	0.02277204
Ridges						
	ER	-	-	-96.517569	195.0470	0.00272541
	ER	λ	0.983126	-96.462497	196.9607	0.00104683
	ER	δ	2.640622	-95.539234	195.1142	0.00263539
	ER	κ	0.711641	-95.925549	195.8868	0.00179089
	SYM	-	-	-96.517569	195.0470	0.00272541
	SYM	λ	0.983126	-96.462497	196.9607	0.00104683
	SYM	δ	2.640623	-95.539234	195.1142	0.00263539

	SYM	κ	0.711641	-95.925549	195.8868	0.00179089
	ARD	-	-	-90.359043	184.7538	0.46835241
	ARD	λ	1.000000	-90.359043	186.7897	0.16922975
	ARD	δ	0.798659	-90.315332	186.7023	0.17679105
	ARD	κ	1.000000	-90.359043	186.7897	0.16922975
Life Habitat						
	ER	-	-	-340.434615	682.8811	0.00000000
	ER	λ	0.999488	-340.433567	684.9030	0.00000000
	ER	δ	1.210208	-340.321251	684.6783	0.00000000
	ER	κ	0.684769	-338.275509	680.5868	0.00000000
	SYM	-	-	-299.291318	643.5067	0.36872043
	SYM	λ	1.000000	-299.291318	645.7953	0.11741502
	SYM	δ	0.664207	-298.962033	645.1368	0.16320389
	SYM	κ	0.762352	-298.197214	643.6071	0.35066054
	ARD	-	-	-289.582448	675.4090	0.00000004
	ARD	λ	0.999811	-289.582310	678.0354	0.00000001
	ARD	δ	0.784813	-289.459821	677.7904	0.00000001
	ARD	κ	0.731187	-288.124860	675.1205	0.00000005
