# Supplementary material for: Phylogenetic mapping of scale nanostructure diversity in snakes
Source: BMC Evol Biol. 2019 Apr 16;19:91. doi: 10.1186/s12862-019-1411-6 (PMC6469093; doi:10.1186/s12862-019-1411-6)
Supplement: Supplementary file 5 — Figure S1. Stochastic mapping of the Cell Shape character on the full species tree. Green, ‘wide’; red, ‘polygonal’. Higher-level taxa are indicated with different colours on the corresponding branches. (PDF 1749 kb) [file 12862_2019_1411_MOESM5_ESM.pdf]

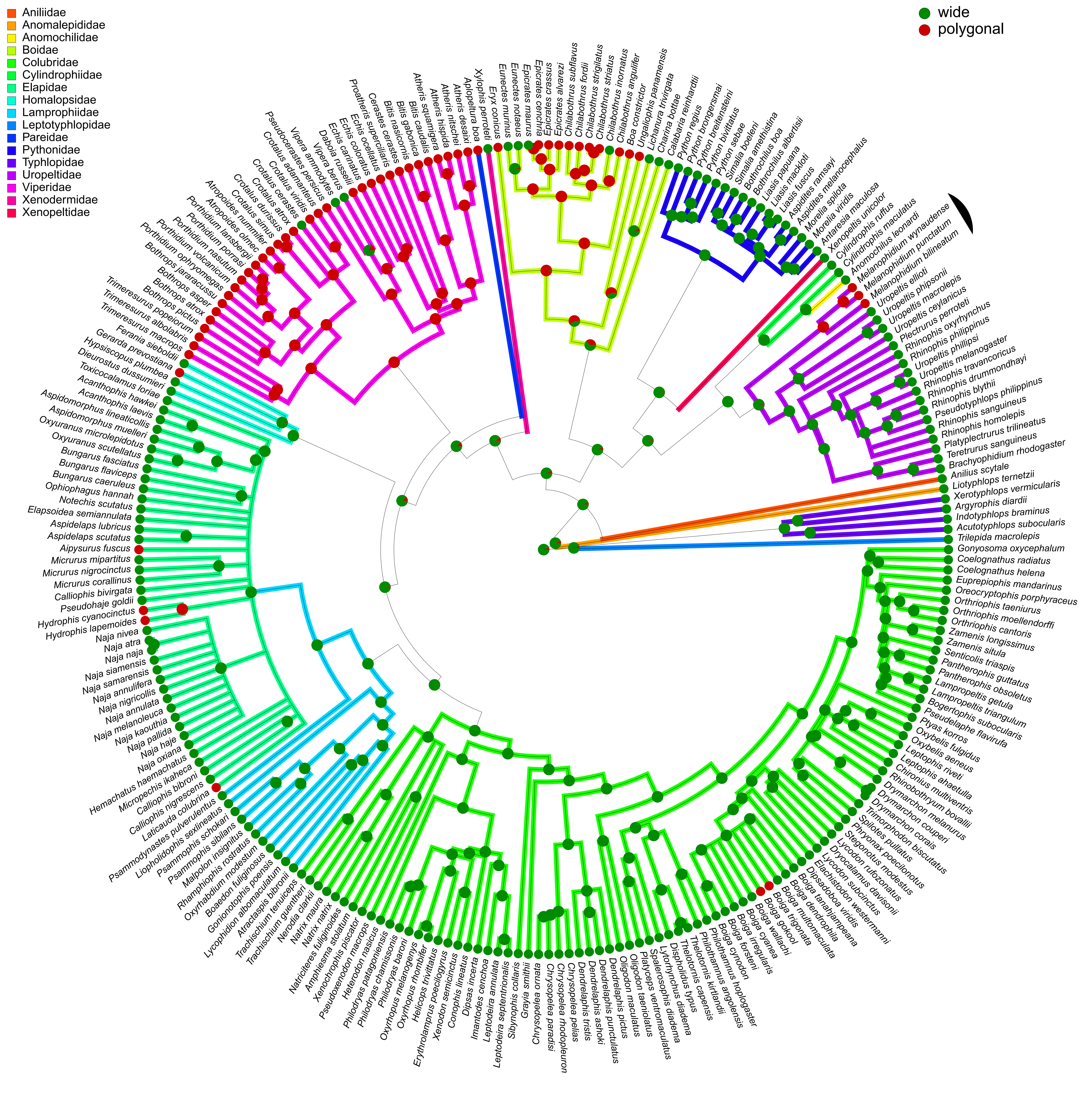

Aniliidae
Anomalepididae
Anomochilidae
Boidae
Colubridae
Cylindrophiidae
Elapidae
Homalopsidae
Lamprophiidae
Leptotyphlopidae
Pareidae
Pythonidae
Typhlopidae
Uropeltidae
Viperidae
Xenodermidae
Xenopeltidae
wide
polygonal
Xylophis perroteti
Aplopeltura boa
Atheris desaixi
Atheris nitschei
Atheris hispida
Atheris squamigera
Bitis caudalis
Bitis gabonica
Bitis nasicornis
Cerastes cerastes
Proatheris superciliaris
Echis ocellatus
Echis coloratus
Echis carinatus
Daboia russelii
Vipera berus
Vipera ammodytes
Pseudocerastes persicus
Crotalus adamanteus
Crotalus viridis
Crotalus cerastes
Crotalus atrox
Crotalus durissus
Crotalus simus
Atropoides nummifer
Atropoides olmec
Porthidium lansbergii
Porthidium porrasi
Porthidium nasutum
Porthidium volcanicum
Porthidium ophryomegas
Bothrops jararacussu
Bothrops asper
Bothrops atrox
Bothrops pictus
Trimeresurus popeiorum
Trimeresurus albolabris
Trimeresurus macrops
Fernania sieboldii
Gerarda prevostiana
Hypsiscopus plumbea
Dieurostus dussumieri
Toxicocalamus loriae
Acanthophis hawkei
Acanthophis laevis
Aspidomorphus lineaticollis
Aspidomorphus muelleri
Oxyuranus microlepidotus
Oxyuranus scutellatus
Bungarus fasciatus
Bungarus flaviceps
Bungarus caeruleus
Ophiophagus hannah
Notechis scutatus
Elapsoidea semiannulata
Aspidelaps lubricus
Aspidelaps scutatus
Aipysurus fuscus
Micrurus mipartitus
Micrurus nigrocinctus
Micrurus corallinus
Calliophis bivirgata
Pseudohaje goldii
Hydrophis cyanocinctus
Hydrophis lapemoides
Naja nivea
Naja atra
Naja naja
Naja siamensis
Naja samarensis
Naja annulifera
Naja nigricollis
Naja annulata
Naja melanoleuca
Naja kaouthia
Naja pallida
Naja haje
Naja oxiana
Hemachatus haemachatus
Micropechis ikaheca
Calliophis bibroni
Calliophis nigrescens
Laticauda colubrina
Psammodynastes pulverulentus
Liopholidophis sexlineatus
Psammophis schokari
Psammophis sibilans
Malpolon insignitus
Rhamphiophis rostratus
Oxyrhabdium modestum
Boaedon fuliginosus
Gonionotophis poensis
Lycophidion albomaculatum
Atractaspis bibronii
Trachischium tenuiceps
Trachischium guentheri
Nerodia clarkii
Natrix maura
Natrix natrix
Natriciteres fuliginoides
Amphiesma stolatum
Xenochrophis piscator
Pseudoxenodon macrops
Heterodon nasicus
Philodryas patagoniensis
Philodryas chamissonis
Philodryas baroni
Oxyrhopus melanogenys
Oxyrhopus rhombifer
Helicops trivittatus
Erythrolamprus poecilogyrus
Xenodon semicinctus
Conophis lineatus
Dipsas incerta
Imantodes cenchoa
Leptodeira annulata
Leptodeira septentrionalis
Sibynophis collaris
Grayia smithii
Chrysopelea ornata
Chrysopelea paradisi
Chrysopelea rhodopleuron
Chrysopelea pelias
Dendrelaphis tristis
Dendrelaphis ashoki
Dendrelaphis punctulatus
Dendrelaphis pictus
Oligodon maculatus
Oligodon taeniolatus
Platyceps ventromaculatus
Spalerosophis diadema
Lytorhynchus diadema
Dispholidus typus
Thelotornis capensis
Thelotornis kirtlandii
Philothamnus angolensis
Philothamnus hoplogaster
Boiga cynodon
Boiga forsteni
Boiga irregularis
Boiga cyanea
Boiga wallachi
Boiga gokool
Boiga trigonata
Boiga multomaculata
Boiga dendrophila
Boiga tanahjampeana
Dipsadoboa viridis
Elachistodon westermanni
Lycodon subcinctus
Dryocalamus davisonii
Lycodon rufozonatus
Stegonotus modestus
Phrynonax poecilonotus
Spilotes pullatus
Trimorphodon biscutatus
Drymarchon corais
Drymarchon couperi
Drymarchon melanurus
Rhinobothryum bovallii
Chironius multiventris
Leptophis ahaetulla
Leptophis riveti
Oxybelis aeneus
Oxybelis fulgidus
Ptyas korros
Pseudelaphe flavirufa
Bogertophis subocularis
Lampropeltis triangulum
Lampropeltis getula
Pantherophis obsoletus
Pantherophis guttatus
Senticolis triaspis
Zamenis situla
Zamenis longissimus
Orthriophis cantoris
Orthriophis moellendorffi
Orthriophis taeniurus
Oreocryptophis porphyraceus
Euprepiophis mandarinus
Coelognathus helena
Coelognathus radiatus
Gonyosoma oxycephalum
Trilepida macrolepis
Acutotyphlops subocularis
Indotyphlops braminus
Argyrophis diardii
Xerotyphlops vermicularis
Liotyphlops ternetzii
Anilius scytale
Brachyophidium rhodogaster
Teretrurus sanguineus
Platyplectrurus trilineatus
Rhinophis homolepis
Rhinophis sanguineus
Pseudotyphlops philippinus
Rhinophis blythii
Rhinophis drummondhayi
Rhinophis travancoricus
Uropeltis melanogaster
Uropeltis phillipsi
Rhinophis philippinus
Rhinophis oxyrhynchus
Plectrurus perroteti
Uropeltis ceylanicus
Uropeltis macrolepis
Uropeltis phipsonii
Uropeltis ellioti
Melanophidium bilineatum
Melanophidium punctatum
Melanophidium wynaudense
Anomochilus leonardi
Cylindrophis maculatus
Cylindrophis ruffus
Xenopeltis unicolor
Antaresia maculosa
Morelia viridis
Morelia spilota
Aspidites melanocephalus
Aspidites ramsayi
Liasis fuscus
Liasis mackloti
Liasis papuana
Bothrochilus albertisii
Bothrochilus boa
Simalia amethistina
Simalia boeleni
Python sebae
Python bivittatus
Python breitensteini
Python brongersmai
Python regius
Calabaria reinhardtii
Charina bottae
Lichanura trivirgata
Ungaliophis panamensis
Boa constrictor
Chilabothrus angulifer
Chilabothrus inornatus
Chilabothrus striatus
Chilabothrus strigilatus
Chilabothrus fordii
Chilabothrus subflavus
Epicrates alvarezi
Epicrates crassus
Epicrates cenchria
Epicrates maurus
Eunectes notaeus
Eunectes murinus
Eryx conicus
