# Supplementary figures and images for: Phylogenetic mapping of scale nanostructure diversity in snakes
Source: BMC Evol Biol. 2019 Apr 16;19:91. doi: 10.1186/s12862-019-1411-6 (PMC6469093; doi:10.1186/s12862-019-1411-6)

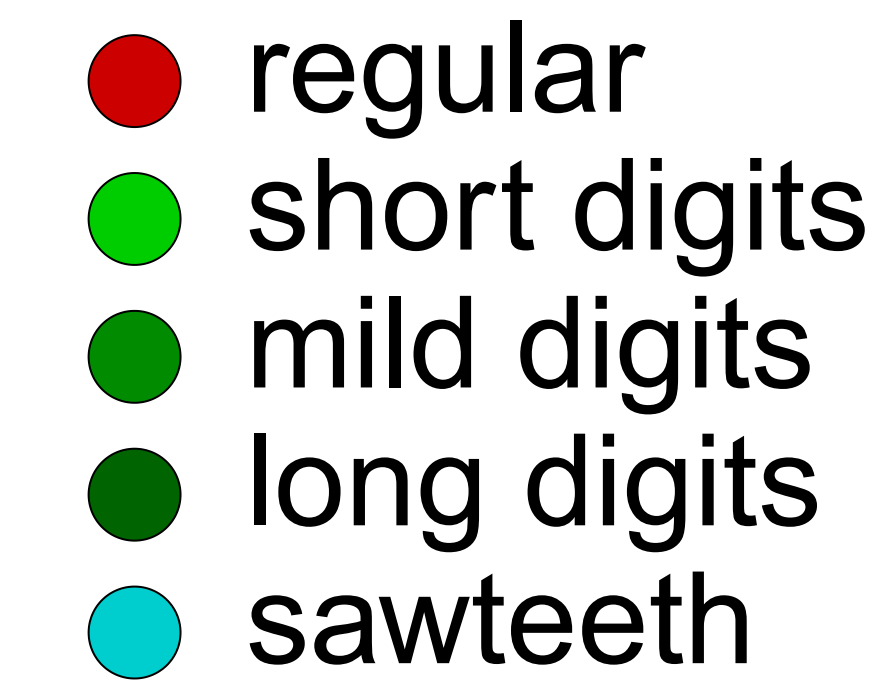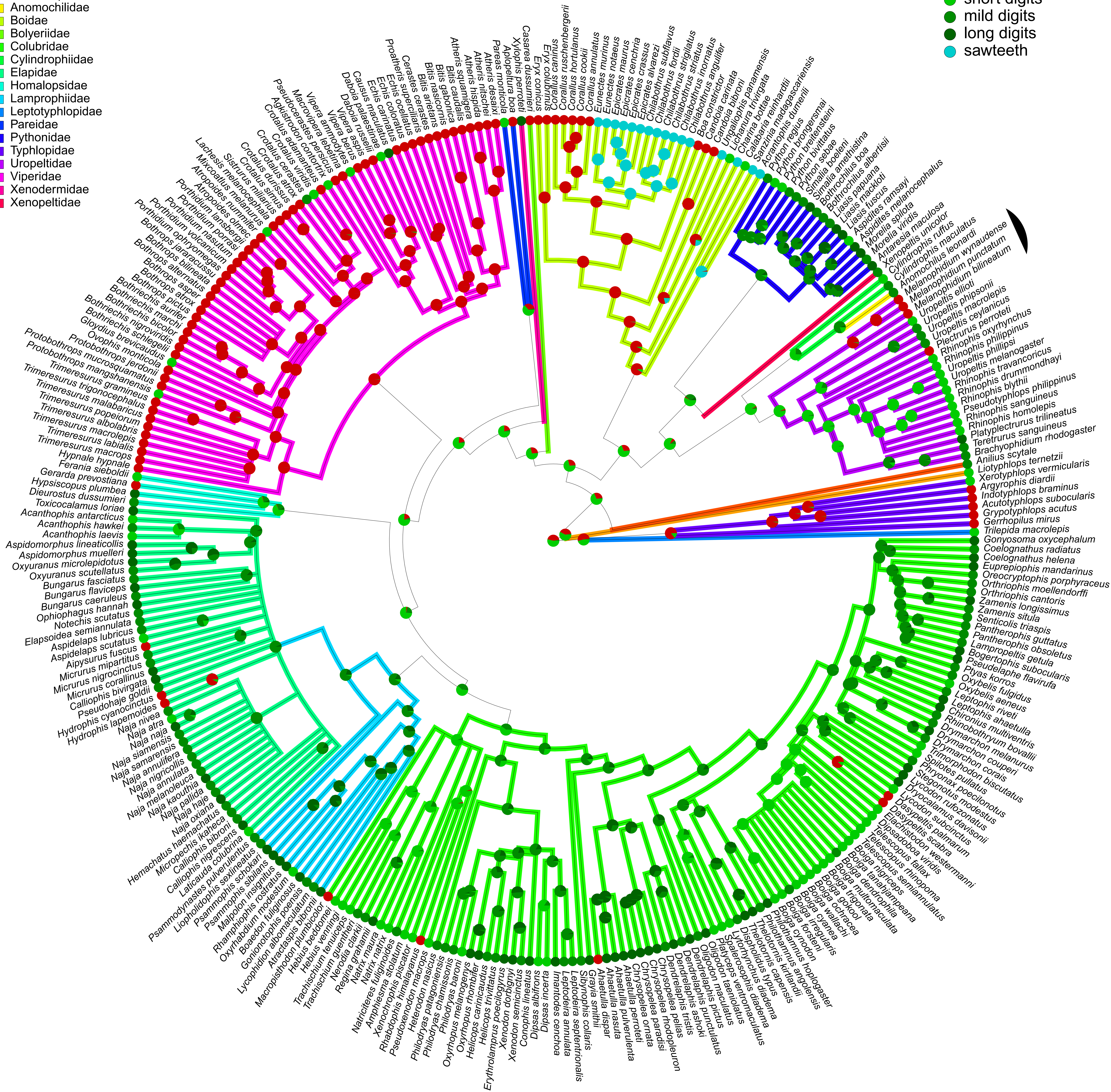

Supplement: Supplementary file 6 — Figure S2. Stochastic mapping of the Cell Border character on the full species tree. Red, ‘regular’; light green, ‘short digits’; mild green, ‘mild digits’; dark green, ‘long digits’; blue, ‘sawteeth’. Higher-level taxa are indicated with different colours on the corresponding branches. (PDF 2299 kb) [file 12862_2019_1411_MOESM6_ESM.pdf]

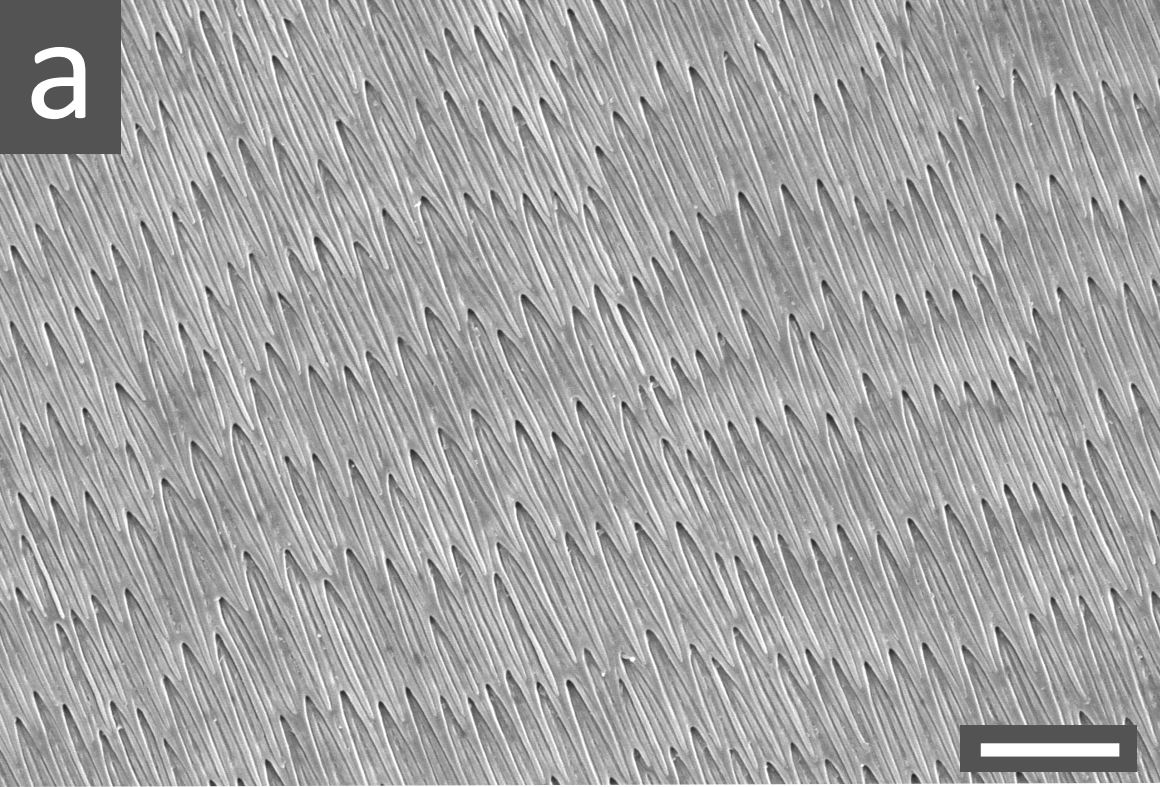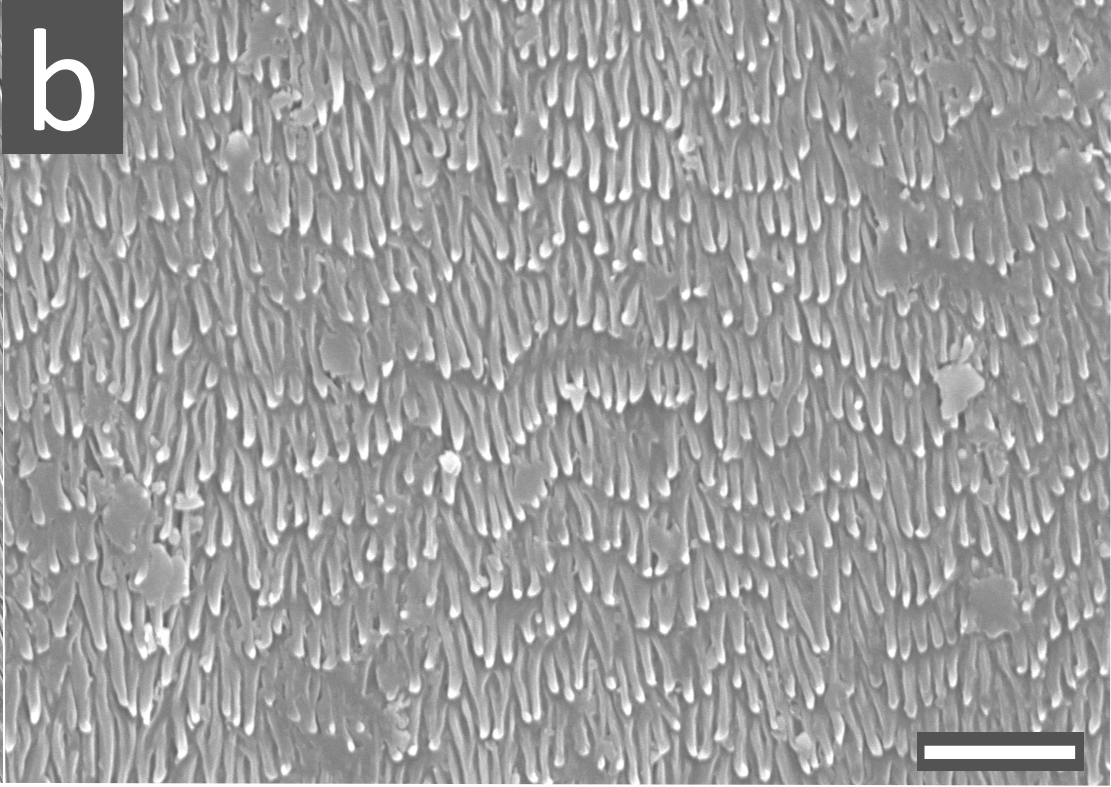

Supplement: Supplementary file 7 — Figure S3. Comparison between two types of digitations. Digits with (a) sharp tips in Boaedon fuliginosus (a representative species of Caenophidia) and (b) round tips in Morelia spilota spilota (a representative of Pythonidae). Scale bars: 5 μm. (PDF 8187 kb) [file 12862_2019_1411_MOESM7_ESM.pdf]

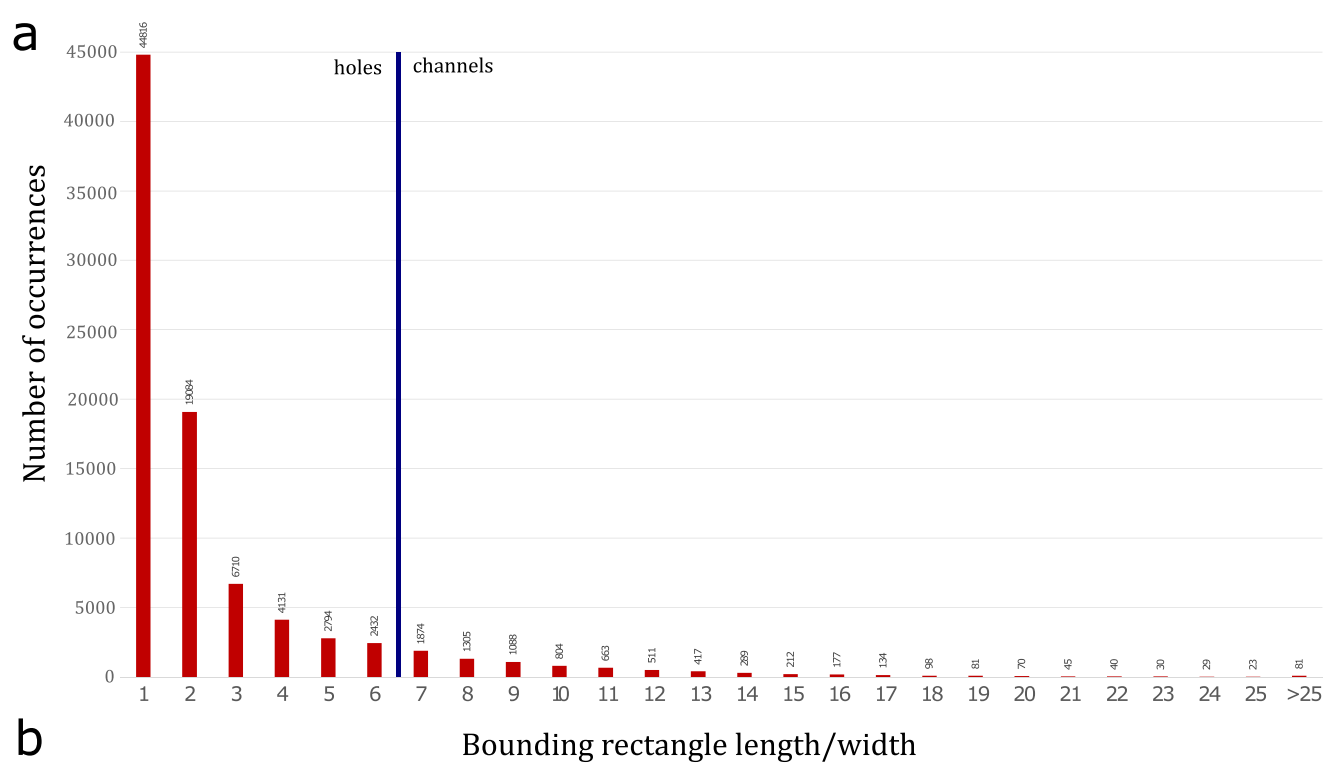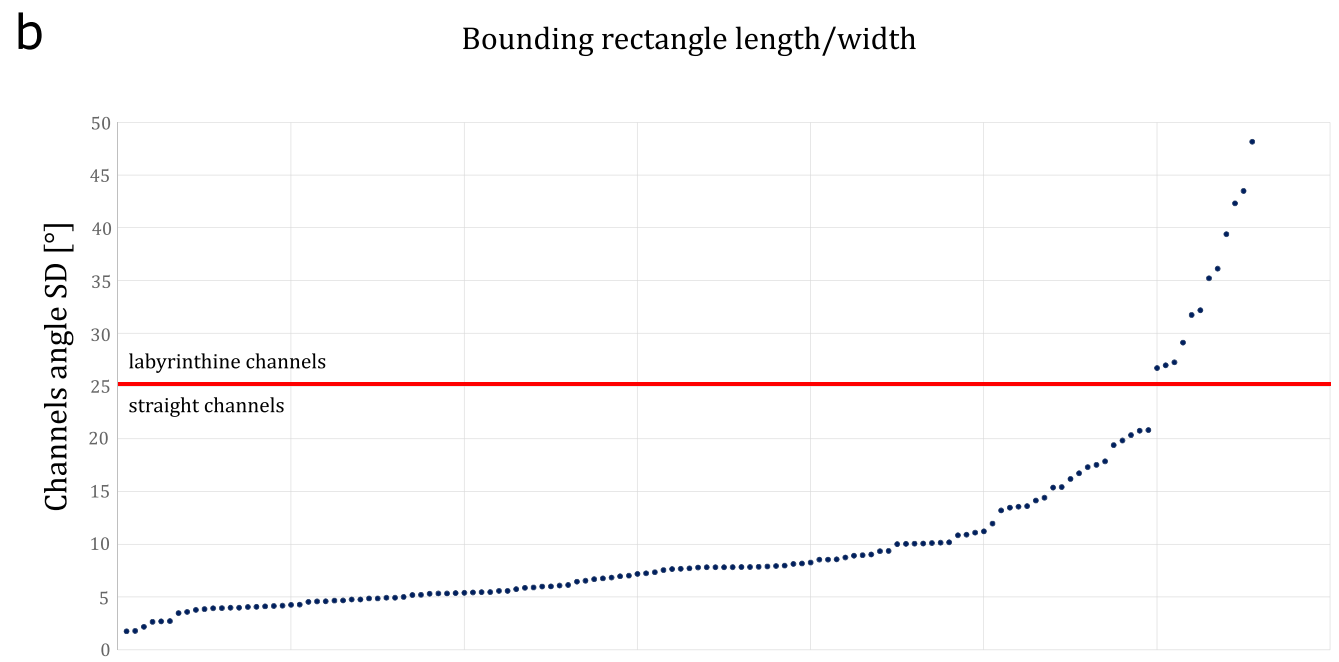

Supplement: Supplementary file 9 — Figure S5. Parameter distributions across all species for categorisation of cell surface structures. (a) Distribution of bounding rectangle length/width ratio (rounded at the nearest integer) for the differentiation of holes and channels. Vertical blue line: arbitrary threshold. (b) Channel angle sorted standard deviations. The chosen threshold to differentiate straight from labyrinthine channels is set at 25° (red line), i.e., within the largest interval of unobserved SD values. (PDF 158 kb) [file 12862_2019_1411_MOESM9_ESM.pdf]

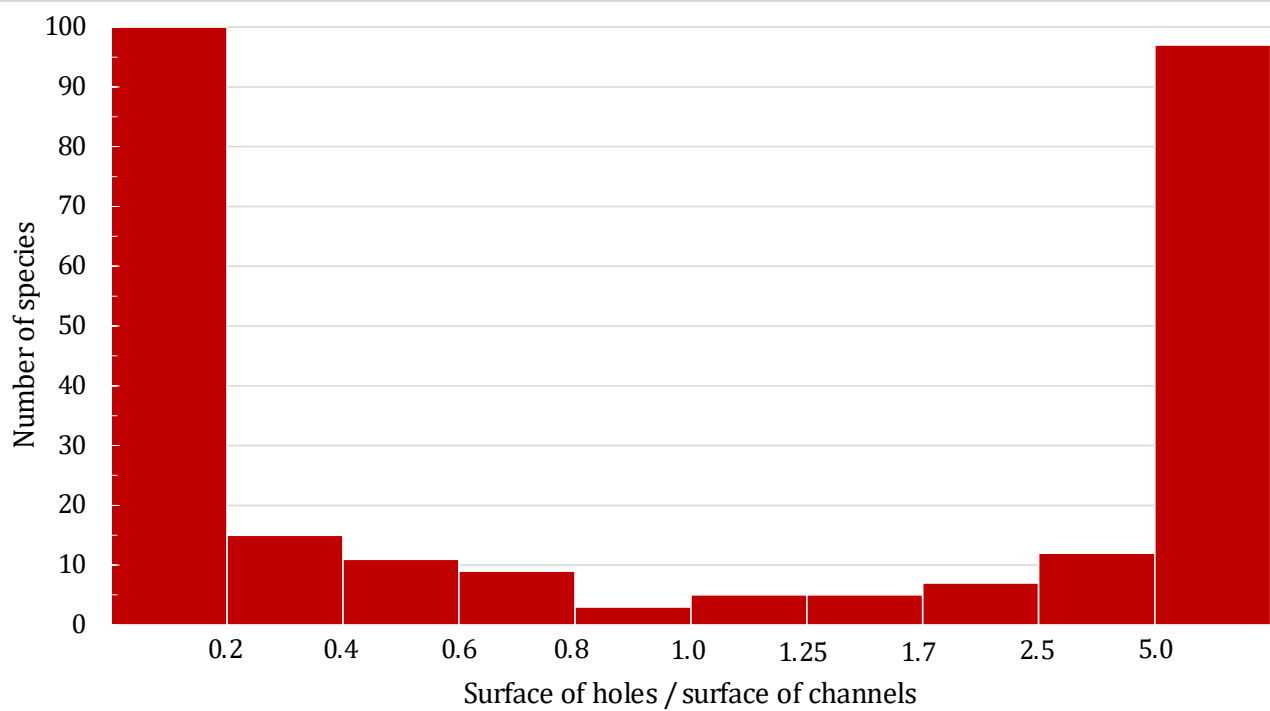

Supplement: Supplementary file 10 — Figure S6. Cell surface state distribution. Distribution across species of the ratios between the surfaces of ‘holes’ and ‘straight channels’ observed within a species. The threshold separating ‘straight channels’ and ‘holes’ corresponds to a ratio of 1.0. (PDF 21 kb) [file 12862_2019_1411_MOESM10_ESM.pdf]

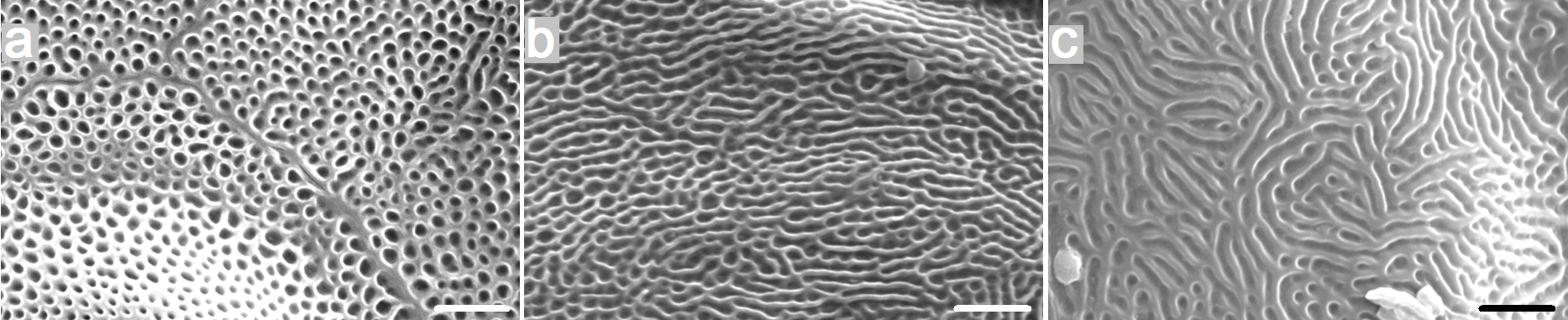

Supplement: Supplementary file 11 — Figure S7. Assignment of the cell surface state. (a) ‘holes’; (b) ambiguity between ‘holes’ and ‘labyrinthine channels’; (c) ‘labyrinthine channels’. Scale bars: 2 μm. (PNG 721 kb) [file 12862_2019_1411_MOESM11_ESM.png]

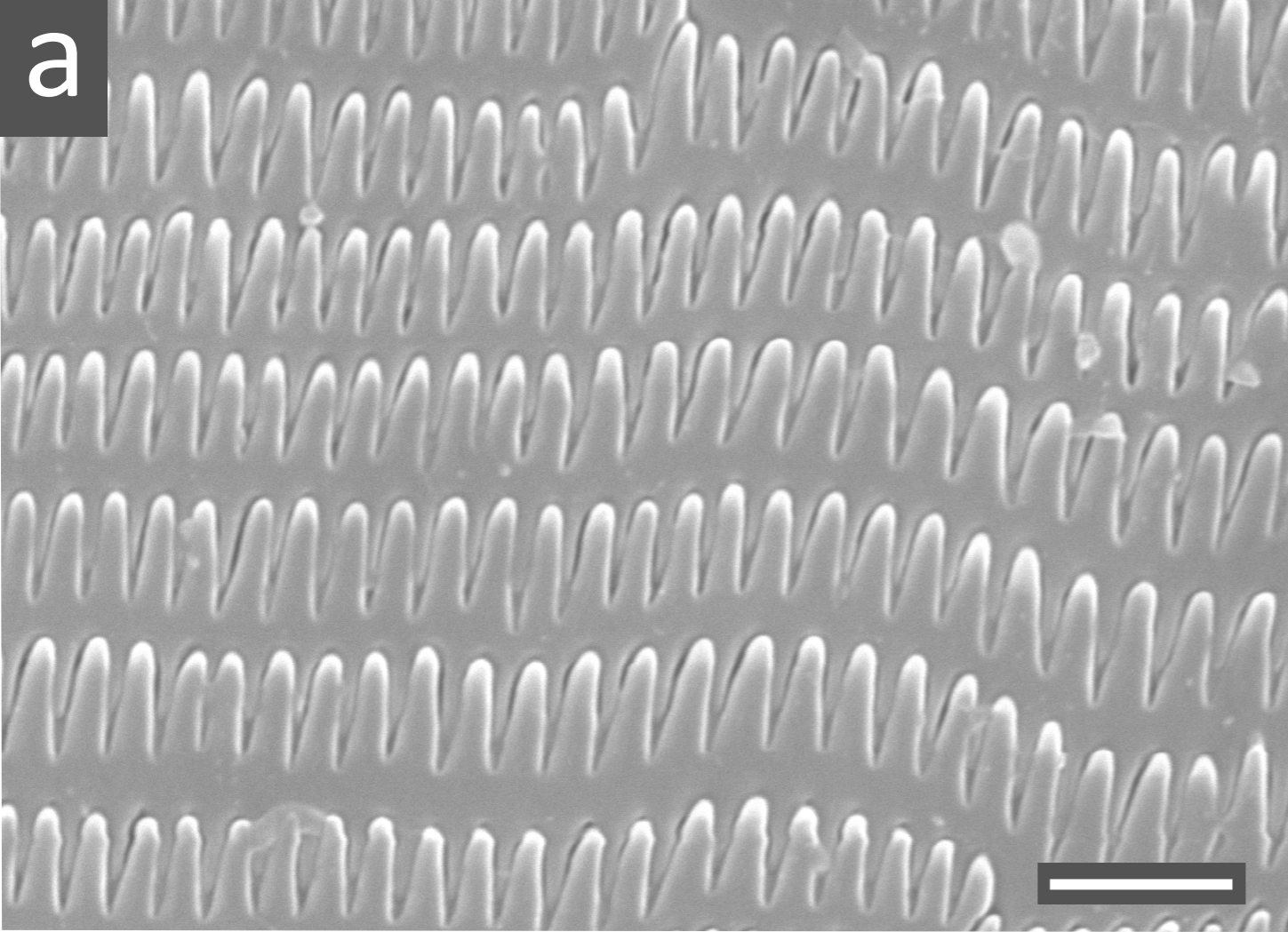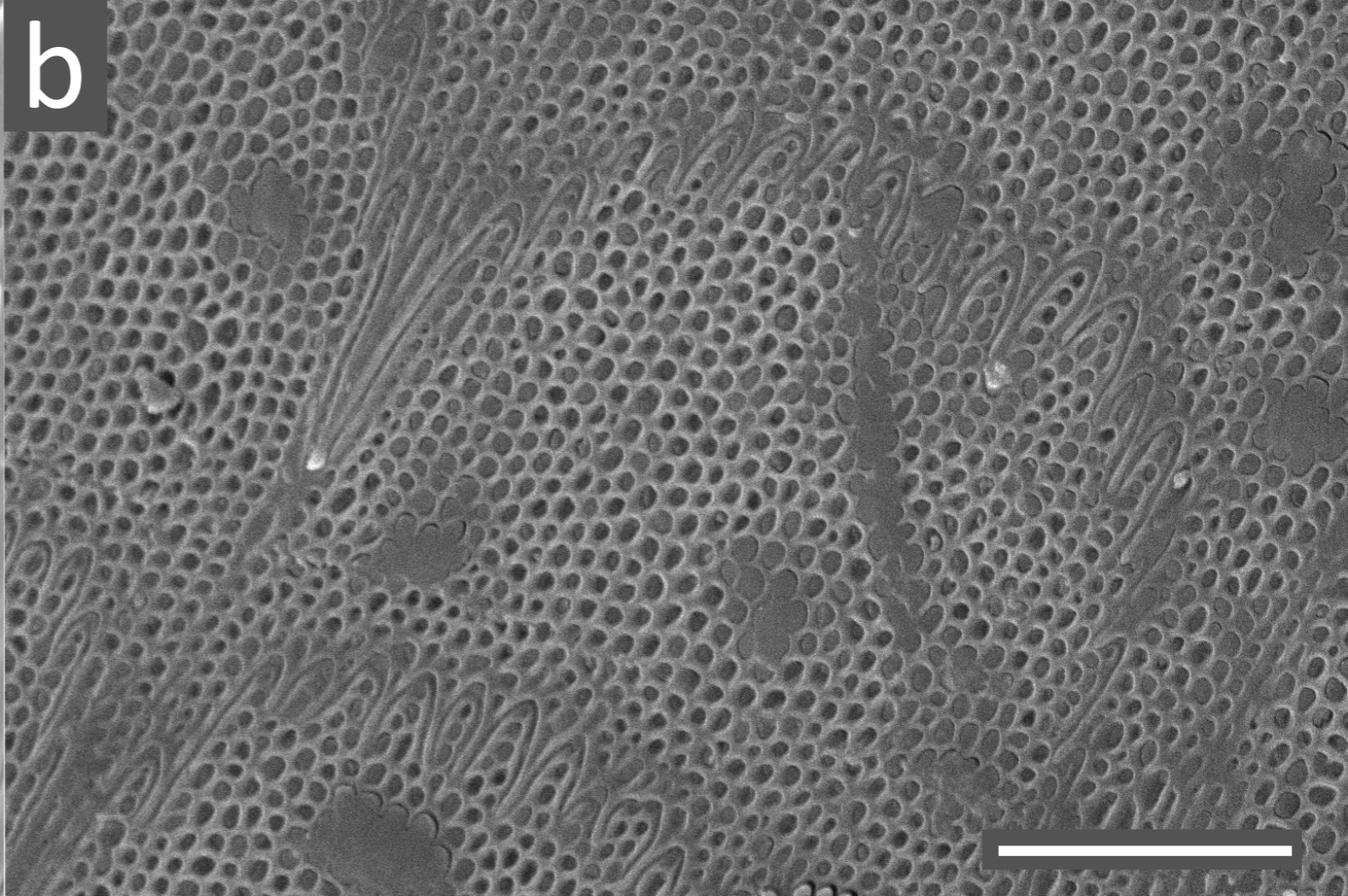

Supplement: Supplementary file 14 — Figure S10. Highly-organised nanostructures. (a) Digitations in Xenopeltis unicolor and (b) cell surface ‘holes’ in Boiga multimaculata. Scale bars: 2 μm (a) and 5 μm (b). (PDF 4238 kb) [file 12862_2019_1411_MOESM14_ESM.pdf]

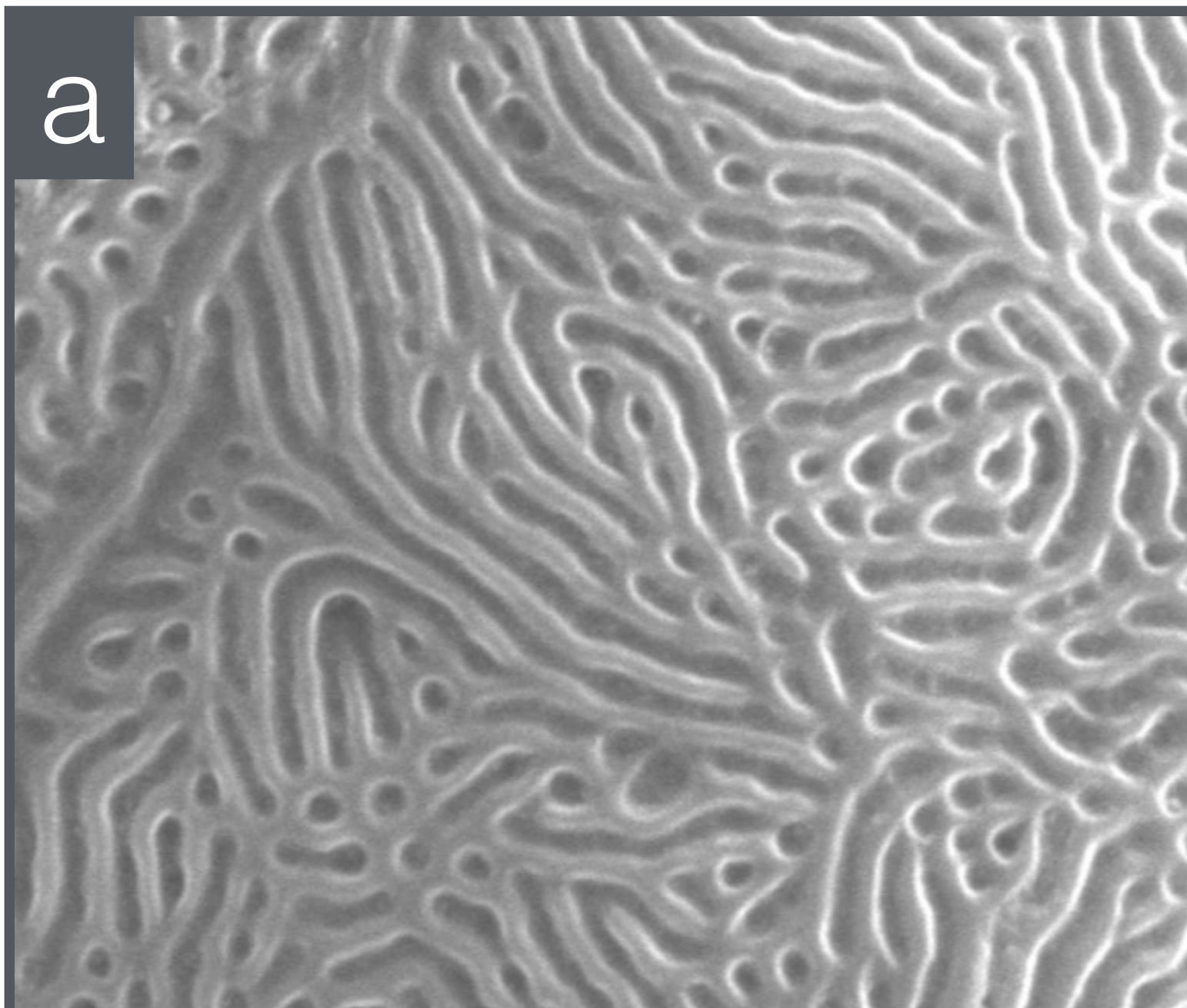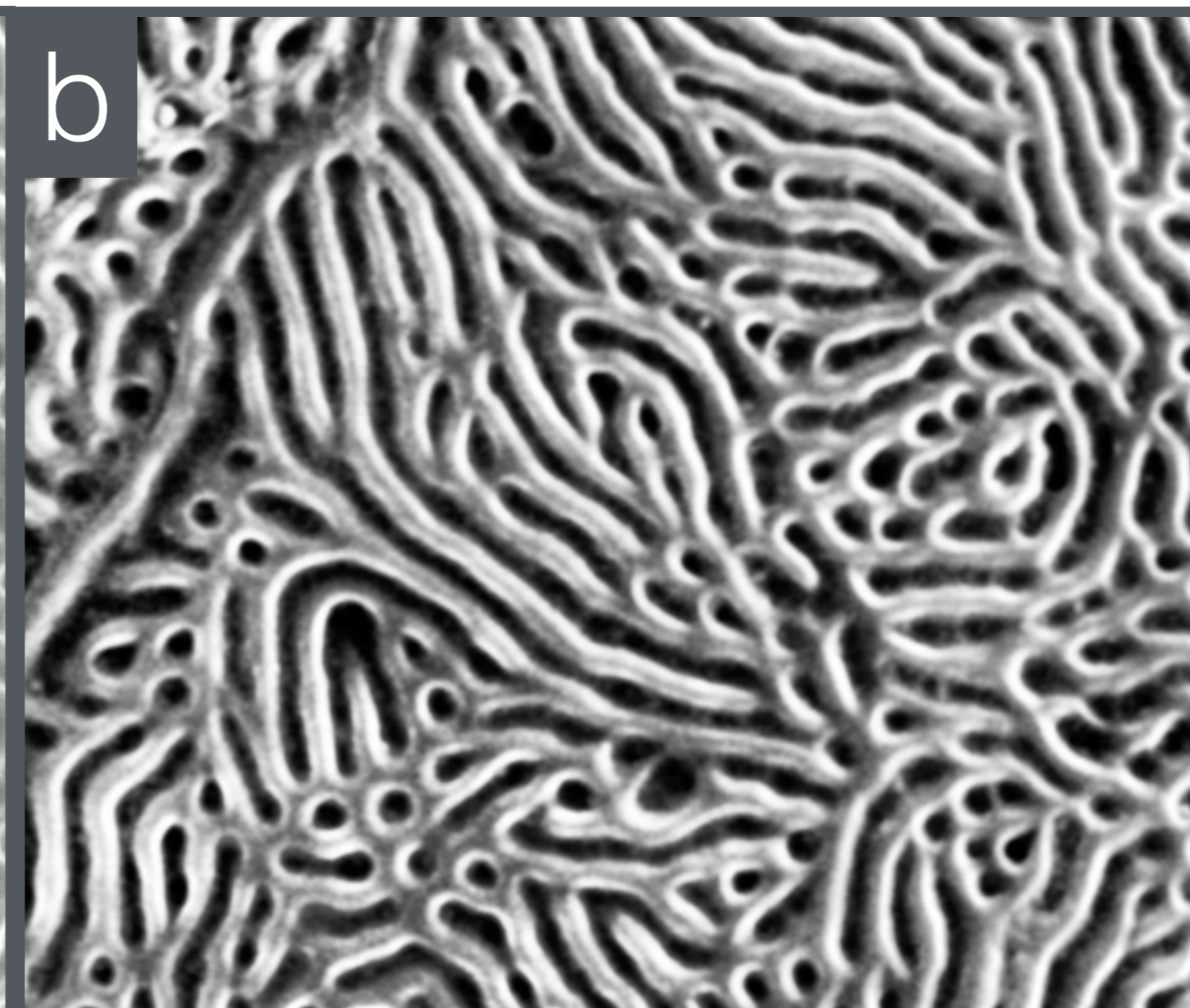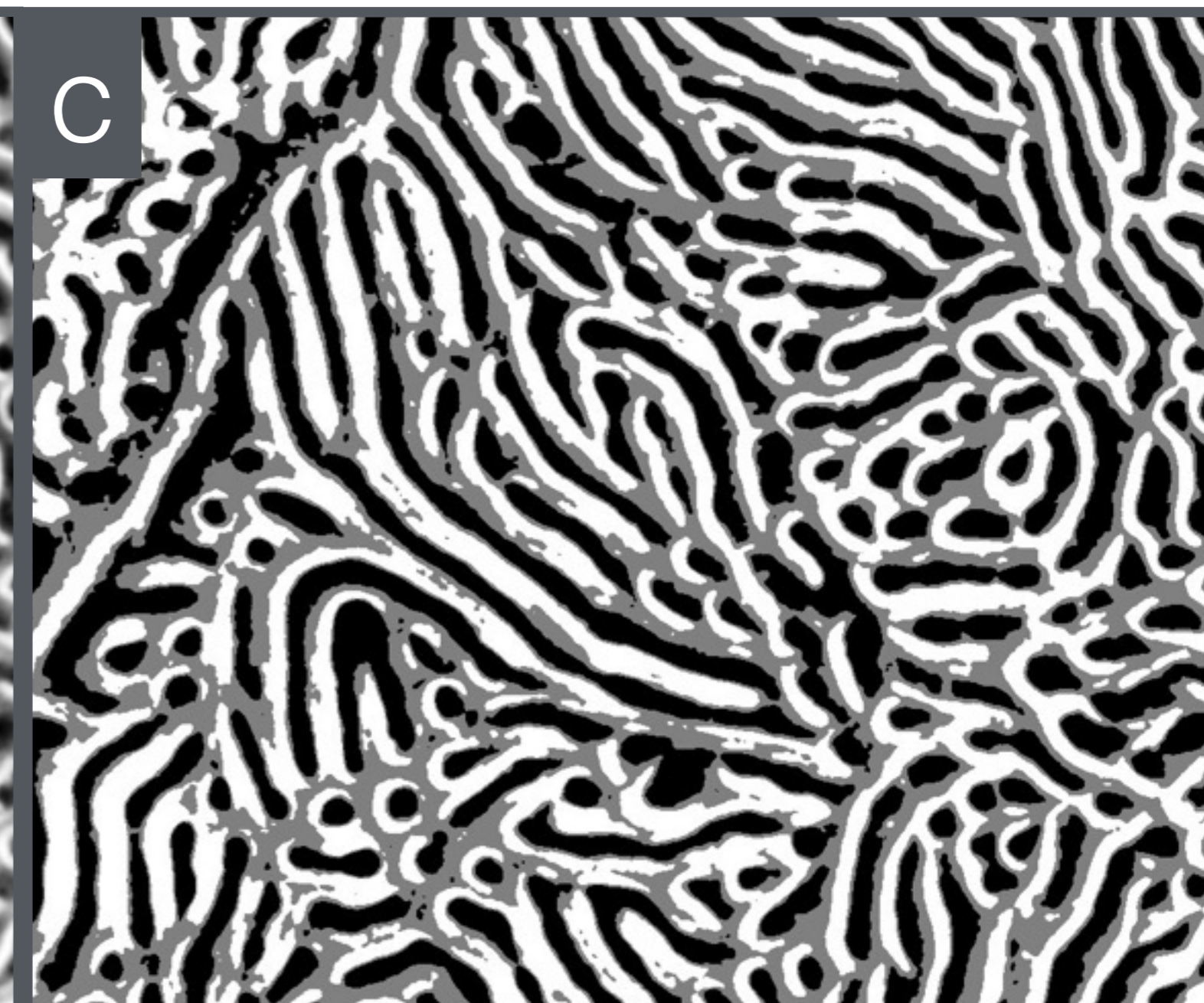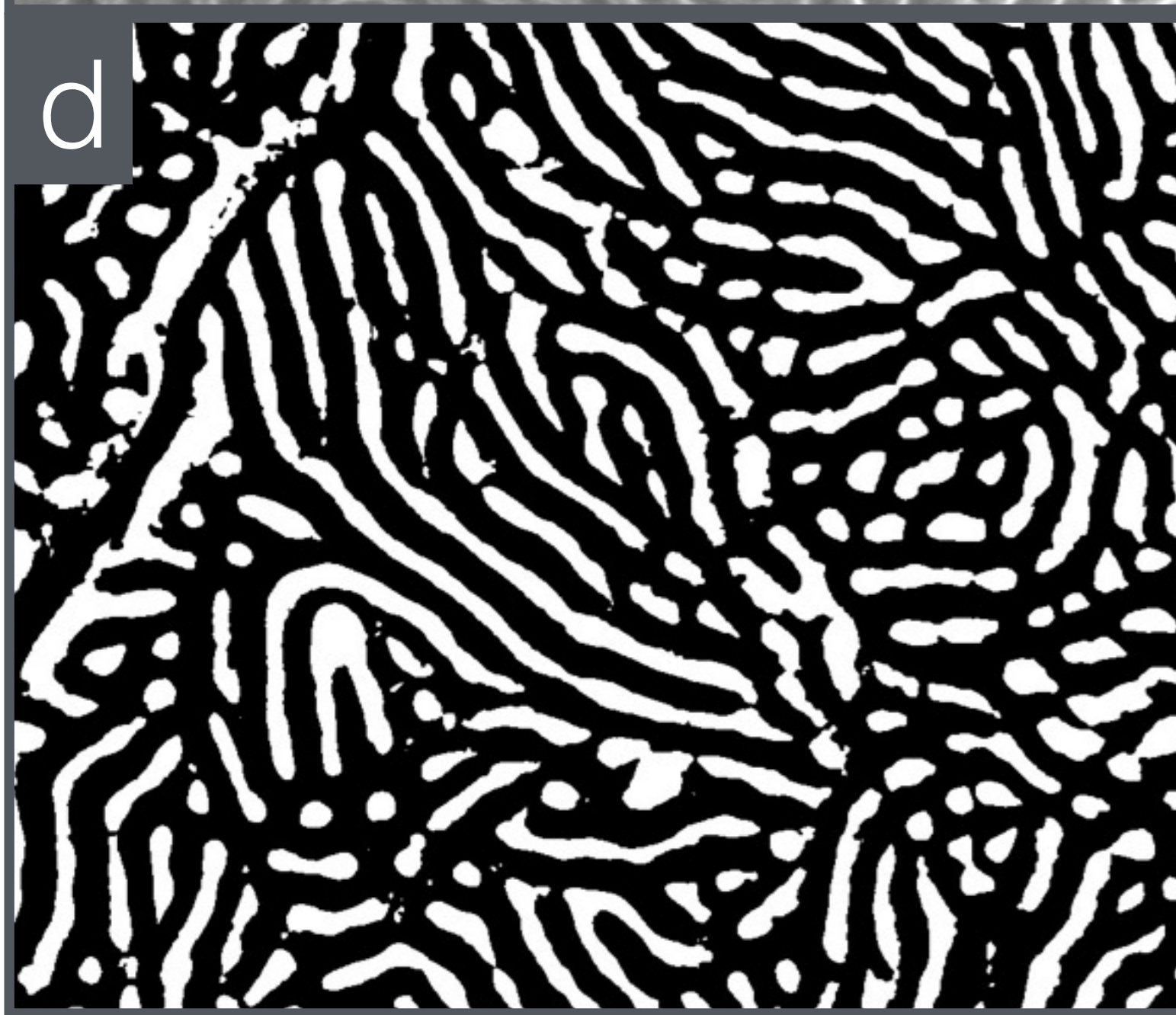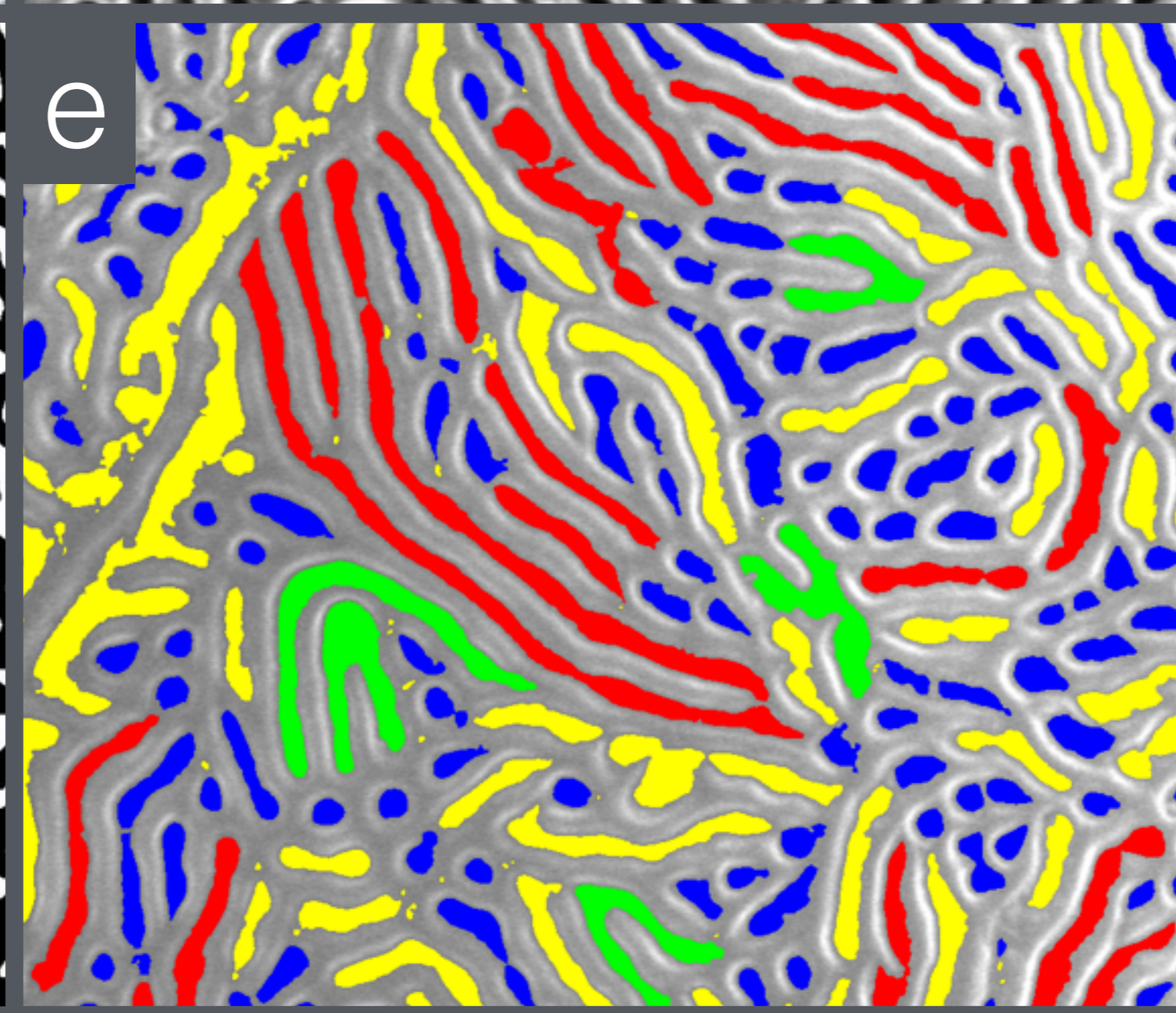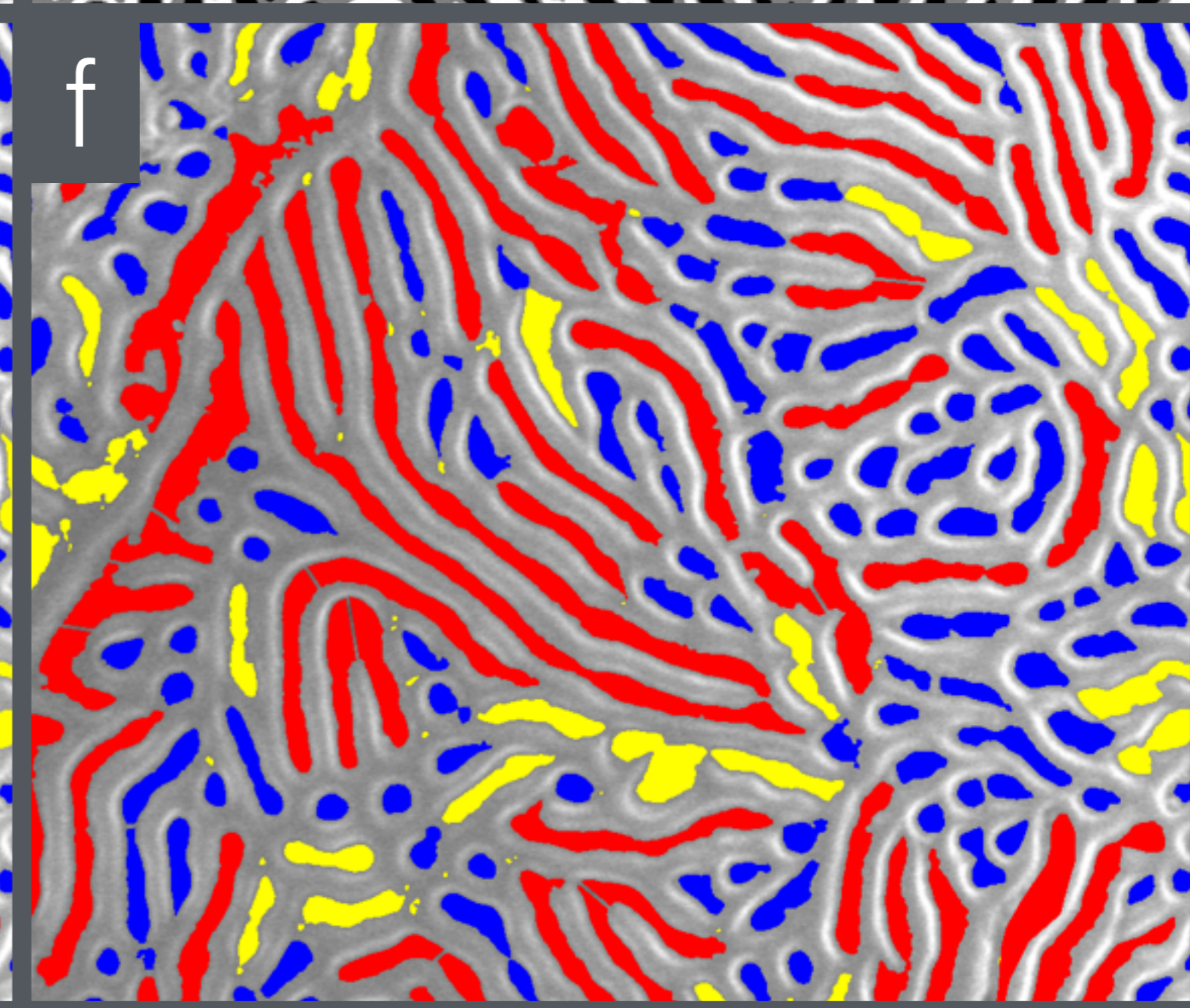

Supplement: Supplementary file 15 — Figure S11. Identification of cell surface structures using image analysis. (a) Original SEM image; (b) Contrast Limited Adaptive Histogram Equalisation; (c) local k-means pixel clustering into three categories based on their intensity (black, grey and white); (d) identification of the darkest pixels that form contours; (e) grouping the contours into four classes: holes (blue), straight channels (red), labyrinthine channels (green) and unclassified (yellow); (f) final classification after manual correction. (PDF 2785 kb) [file 12862_2019_1411_MOESM15_ESM.pdf]
