# Supplementary material for: Phylogenetic mapping of scale nanostructure diversity in snakes
Source: BMC Evol Biol. 2019 Apr 16;19:91. doi: 10.1186/s12862-019-1411-6 (PMC6469093; doi:10.1186/s12862-019-1411-6)
Supplement: Supplementary file 8 — Figure S4. Stochastic mapping of the Cell Surface character on the full species tree. Red, ‘smooth’; green, ‘holes’; blue, ‘straight channels’; yellow, ‘labyrinthine channels’. Asterisks indicate species categorised as ‘smooth’ although they possess very small depressions that do not pass the algorithm’s requirements to be classified as ‘holes’. Higher-level taxa are indicated with different colours on the corresponding branches. (PDF 2359 kb) [file 12862_2019_1411_MOESM8_ESM.pdf]

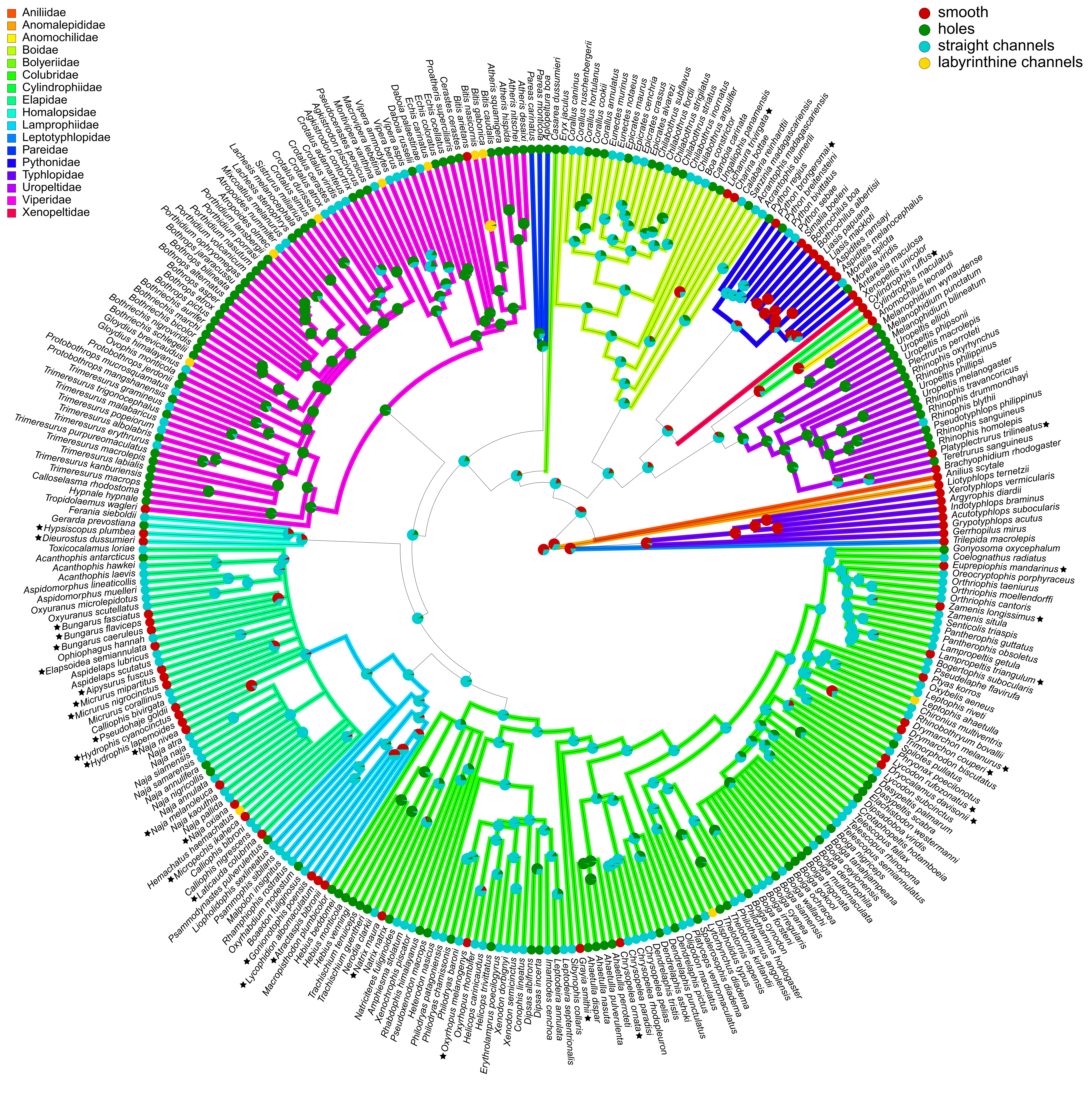

Aniliidae
Anomalepididae
Anomochilidae
Boidae
Bolyeriidae
Colubridae
Cylindrophiidae
Elapidae
Homalopsidae
Lamprophiidae
Leptotyphlopidae
Pareidae
Pythonidae
Typhlopidae
Uropeltidae
Viperidae
Xenopeltidae
smooth
holes
straight channels
labyrinthine channels
Gonyosoma oxycephalum
Coelognathus radiatus
Euprepiophis mandarinus ★
Oreocryptophis porphyraceus
Orthriophis taeniurus
Orthriophis moellendorffi
Orthriophis cantoris
Zamenis longissimus ★
Zamenis situla
Senticolis triaspis
Pantherophis guttatus
Pantherophis obsoletus
Lampropeltis getula
Lampropeltis triangulum ★
Bogertophis subocularis
Pseudelaphe flavirufa
Ptyas korros
Oxybelis aeneus
Leptophis riveti
Leptophis ahaetulla
Chironius multiventris
Rhinobothryum bovallii
Drymarchon melanurus ★
Drymarchon couperi ★
Trimorphodon biscutatus
Spilotes pullatus
Phryonax poecilonotus
Lycodon rufozonatus ★
Dryocalamus davisonii ★
Lycodon subcinctus
Dasypeltis palmarum
Dasypeltis scabra
Elachistodon westermanni
Dipsadoboa viridis
Crotaphopeltis hotamboeia
Telescopus fallax
Telescopus rhinopoma
Telescopus semiannulatus
Boiga nigriceps
Boiga tanahjampeana
Boiga ceylonensis
Boiga dendrophila
Boiga multomaculata
Boiga trigonata
Boiga gokool
Boiga ochracea
Boiga wallachi
Boiga siamensis
Boiga cyanea
Boiga irregularis
Boiga forsteni
Boiga cynodon
Philothamnus hoplogaster
Philothamnus angolensis
Thelotornis kirtlandii
Thelotornis capensis
Dispholidus typus
Lytorhynchus diadema
Spalerosophis diadema
Platyceps ventromaculatus
Oligodon maculatus
Dendrelaphis pictus
Dendrelaphis punctulatus
Dendrelaphis ashoki
Dendrelaphis tristis
Chrysopelea pelias
Chrysopelea rhodopleuron
Chrysopelea paradisi
Chrysopelea ornata ★
Ahaetulla perroteti
Ahaetulla pulverulenta
Ahaetulla nasuta
Ahaetulla dispar
Grayia smithii ★
Sibynophis collaris
Leptodeira septentrionalis
Leptodeira annulata
Imantodes cenchoa
Dipsas incerta
Dipsas albifrons
Conophis lineatus
Xenodon semicinctus
Xenodon dorbignyi
Erythrolamprus poecilogyrus
Helicops trivittatus
Helicops carinicaudus
Oxyrhopus rhombifer
★ Oxyrhopus melanogenys
Philodryas baroni
Philodryas chamissonis
Philodryas patagoniensis
Heterodon nasicus
Pseudoxenodon macrops
Rhabdophis himalayanus
Xenochrophis piscator
Amphiesma stolatum
Natriciteres fuliginoides
Natrix natrix
★ Natrix maura
Nerodia clarkii
Trachischium guentheri
Trachischium tenuiceps
Hebius venningi
Hebius monticola
Hebius beddomei
Macropisthodon plumbicolor
★ Atractaspis bibronii
★ Lycophidion albomaculatum
★ Gonionotophis poensis
Boaedon fuliginosus
Oxyrhabdium modestum
Rhamphiophis rostratus
Malpolon insignitus
Psammophis sibilans
Liopholidophis sexlineatus
Psammodynastes pulverulentus
★ Laticauda colubrina
Calliophis nigrescens
Calliophis bibroni
★ Micropechis ikaheca
Hemachatus haemachatus
★ Naja oxiana
Naja pallida
Naja kaouthia
★ Naja melanoleuca
Naja annulata
Naja nigricollis
Naja annulifera
Naja samarensis
Naja siamensis
Naja naja
Naja atra
★ Naja nivea
★ Hydrophis lapemoides
★ Hydrophis cyanocinctus
★ Pseudohaje goldii
Calliophis bivirgata
Micrurus corallinus
★ Micrurus nigrocinctus
★ Micrurus mipartitus
★ Aipysurus fuscus
Aspidelaps scutatus
Aspidelaps lubricus
★ Elapsoidea semiannulata
Ophiophagus hannah
★ Bungarus caeruleus
★ Bungarus flaviceps
★ Bungarus fasciatus
Oxyuranus scutellatus
Oxyuranus microlepidotus
Aspidomorphus muelleri
Aspidomorphus lineaticollis
Acanthophis laevis
Acanthophis hawkei
Acanthophis antarcticus
Toxicocalamus loriae
★ Dieurostus dussumieri
★ Hypsiscopus plumbea
Gerarda prevostiana
Ferania sieboldii
Tropidolaemus wagleri
Hypnale hypnale
Calloselasma rhodostoma
Trimeresurus macrops
Trimeresurus kanburiensis
Trimeresurus labialis
Trimeresurus macrolepis
Trimeresurus purpureomaculatus
Trimeresurus erythrurus
Trimeresurus albolabris
Trimeresurus popeiorum
Trimeresurus malabaricus
Trimeresurus trigonocephalus
Trimeresurus gramineus
Protobothrops mangshanensis
Protobothrops mucrosquamatus
Protobothrops jerdonii
Ovophis monticola
Gloydius himalayanus
Gloydius brevicaudus
Bothriechis schlegelii
Bothriechis nigroviridis
Bothriechis bicolor
Bothriechis marchi
Bothriechis aurifer
Bothrops pictus
Bothrops atrox
Bothrops asper
Bothrops alternatus
Bothrops bilineata
Bothrops jararacussu
Porthidium ophryomegas
Porthidium volcanicum
Porthidium nasutum
Porthidium porrasi
Porthidium lansbergii
Atropoides olmec
Atropoides nummifer
Mixcoatlus melanurus
Lachesis stenophrys
Lachesis melanocephala
Sistrurus miliarius
Crotalus simus
Crotalus durissus
Crotalus atrox
Crotalus cerastes
Crotalus viridis
Crotalus adamanteus
Agkistrodon contortrix
Agkistrodon piscivorus
Pseudocerastes persicus
Montivipera xanthina
Macrovipera lebetina
Vipera ammodytes
Vipera berus
Vipera aspis
Daboia russelii
Daboia palaestinae
Echis carinatus
Echis coloratus
Echis ocellatus
Proatheris superciliaris
Cerastes cerastes
Bitis arietans
Bitis nasicornis
Bitis gabonica
Bitis caudalis
Atheris squamigera
Atheris hispida
Atheris nitschei
Atheris desaixi
Pareas carinatus
Pareas monticola
Aplopeltura boa
Casarea dussumieri
Eryx jaculus
Corallus caninus
Corallus ruschenbergerii
Corallus hortulanus
Corallus cookii
Corallus annulatus
Eunectes murinus
Eunectes notaeus
Epicrates maurus
Epicrates cenchria
Epicrates crassus
Epicrates alvarezi
Chilabothrus subflavus
Chilabothrus fordii
Chilabothrus strigilatus
Chilabothrus striatus
Chilabothrus inornatus
Chilabothrus angulifer
Boa constrictor
Candoia carinata
Ungaliophis panamensis
Lichanura trivirgata ★
Charina bottae
Calabaria reinhardtii
Sanzinia madagascariensis
Acrantophis madagascariensis
Acrantophis dumerili
Python regius
Python brongersmai ★
Python breitensteini
Python bivittatus
Python sebae
Simalia boeleni
Bothrochilus boa
Bothrochilus albertisii
Liasis papuana
Liasis mackloti
Aspidites ramsayi
Aspidites melanocephalus
Morelia spilota
Morelia viridis
Antaresia maculosa
Xenopeltis unicolor
Cylindrophis ruffus ★
Cylindrophis maculatus
Anomochilus leonardi
Melanophidium wynaudense
Melanophidium punctatum
Melanophidium bilineatum
Uropeltis ellioti
Uropeltis phipsonii
Uropeltis macrolepis
Plectrurus perroteti
Rhinophis oxyrhynchus
Rhinophis philippinus
Uropeltis phillipsi
Uropeltis melanogaster
Rhinophis travancoricus
Rhinophis drummondhayi
Rhinophis blythii
Pseudotyphlops philippinus
Rhinophis sanguineus
Rhinophis homolepis
Platyplectrurus trilineatus ★
Teretrurus sanguineus
Brachyophidium rhodogaster
Anilius scytale
Liotyphlops ternetzii
Xerotyphlops vermicularis
Argyrophis diardii
Indotyphlops braminus
Acutotyphlops subocularis
Grypotyphlops acutus
Gerrhopilus mirus
Trilepida macrolepis
