# Supplementary material for: Phylogenetic mapping of scale nanostructure diversity in snakes
Source: BMC Evol Biol. 2019 Apr 16;19:91. doi: 10.1186/s12862-019-1411-6 (PMC6469093; doi:10.1186/s12862-019-1411-6)
Supplement: Supplementary file 12 — Figure S8. Stochastic mapping of the Ridge character on the full species tree. Green, ‘absence’; red, ‘presence’. Higher-level taxa are indicated with different colours on the corresponding branches. (PDF 329 kb) [file 12862_2019_1411_MOESM12_ESM.pdf]

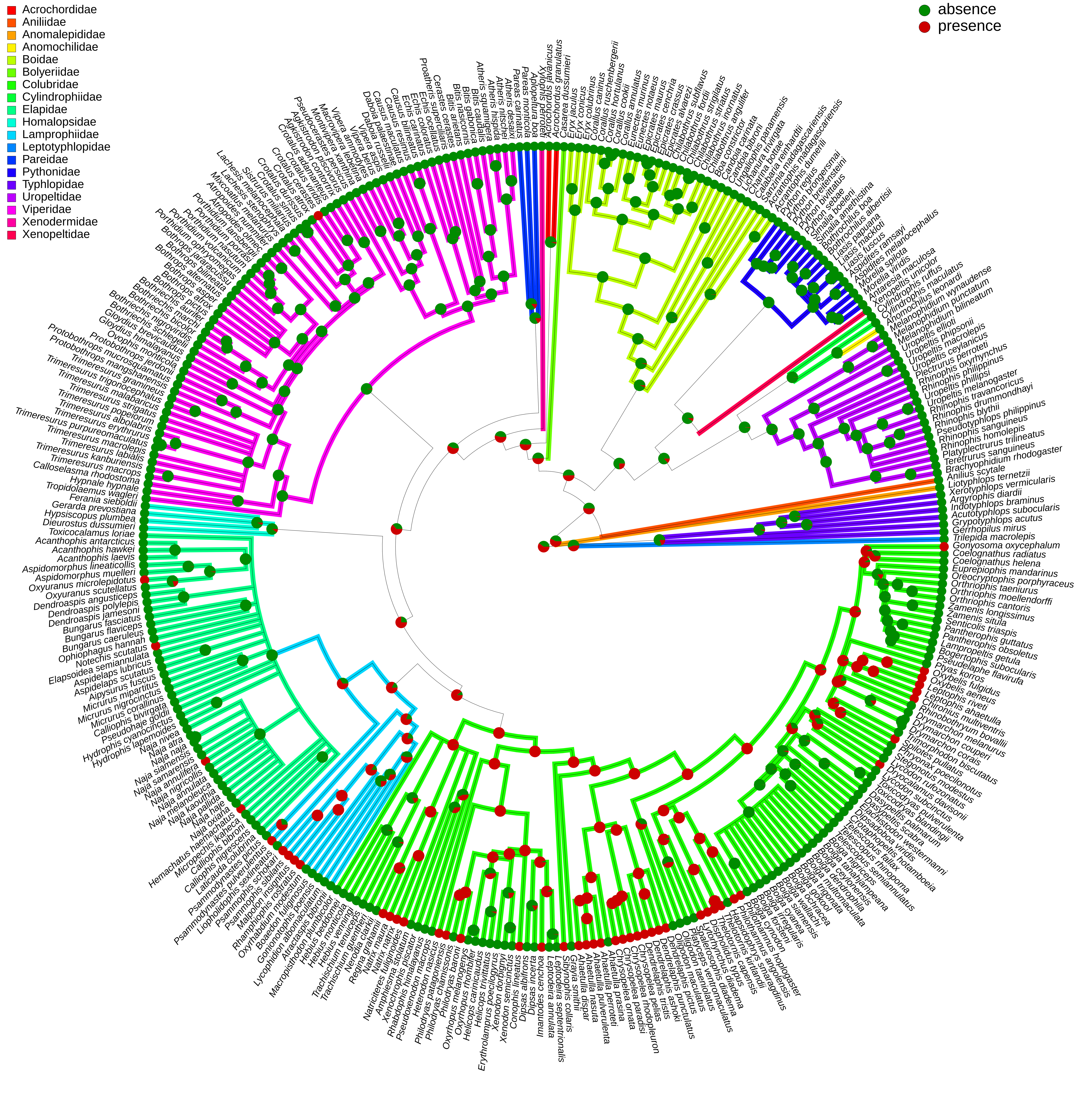

Acrochordidae
Aniliidae
Anomalepididae
Anomochilidae
Boidae
Bolyeriidae
Colubridae
Cylindrophiidae
Elapidae
Homalopsidae
Lamprophiidae
Leptotyphlopidae
Pareidae
Pythonidae
Typhlopidae
Uropeltidae
Viperidae
Xenodermidae
Xenopeltidae
absence
presence
