# Supplementary material for: Phylogenetic mapping of scale nanostructure diversity in snakes
Source: BMC Evol Biol. 2019 Apr 16;19:91. doi: 10.1186/s12862-019-1411-6 (PMC6469093; doi:10.1186/s12862-019-1411-6)
Supplement: Supplementary file 13 — Figure S9. Stochastic mapping of the Life Habit character on the full species tree. Red, ‘aquatic’; dark green, ‘terrestrial’; blue, ‘fossorial’; yellow, ‘arboreal’; orange, ‘aquatic + terrestrial’; turquoise, ‘terrestrial + fossorial’; light green, ‘terrestrial + arboreal’. Higher-level taxa are indicated with different colours on the corresponding branches. (PDF 328 kb) [file 12862_2019_1411_MOESM13_ESM.pdf]

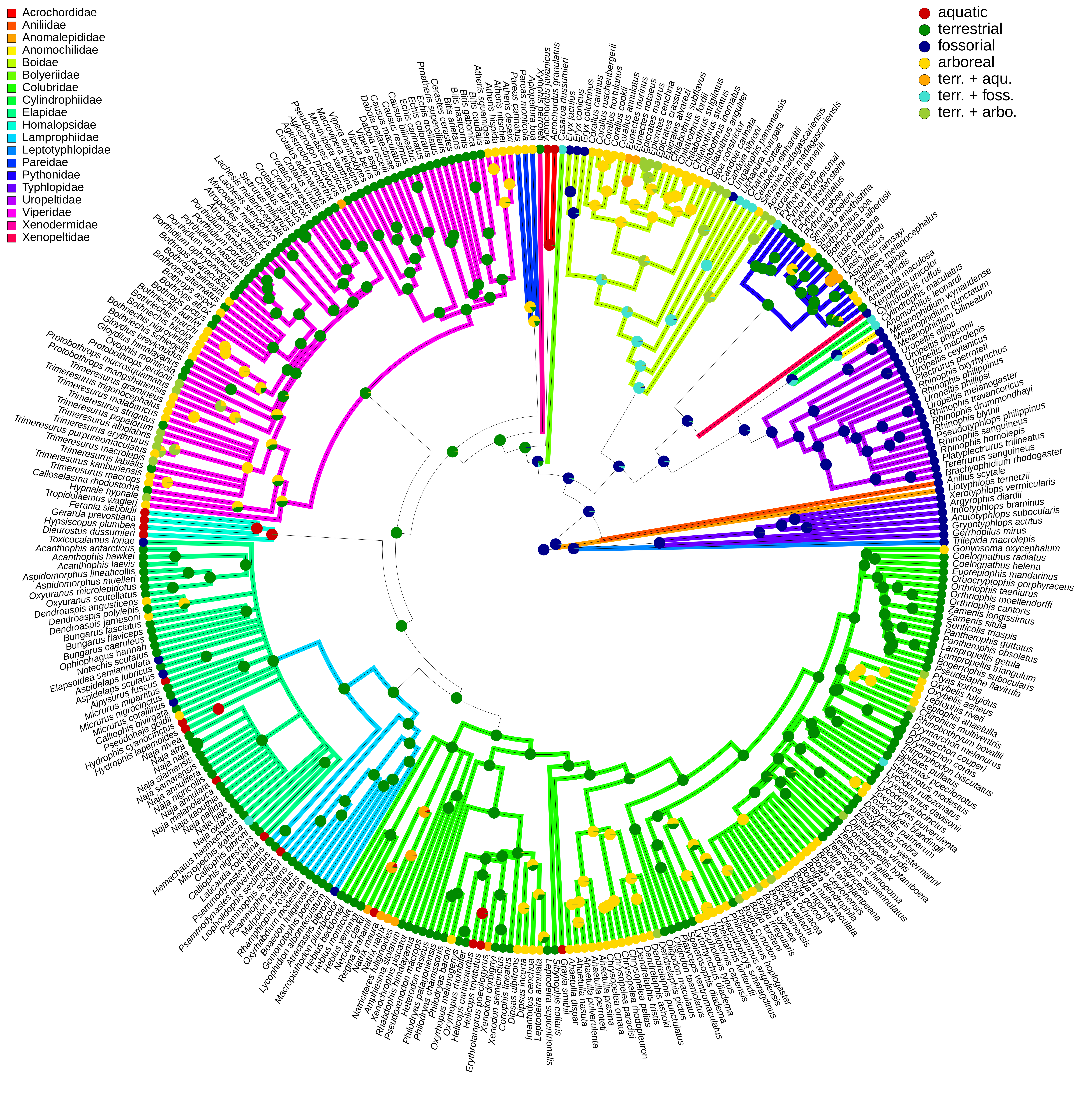

Acrochordidae
Aniliidae
Anomalepididae
Anomochilidae
Boidae
Bolyeriidae
Colubridae
Cylindrophiidae
Elapidae
Homalopsidae
Lamprophiidae
Leptotyphlopidae
Pareidae
Pythonidae
Typhlopidae
Uropeltidae
Viperidae
Xenodermidae
Xenopeltidae
aquatic
terrestrial
fossorial
arboreal
terr. + aqu.
terr. + foss.
terr. + arbo.
